# Non-equilibrium signal integration in hydrogels

**DOI:** 10.1038/s41467-019-14114-0

**Published:** 2020-01-20

**Authors:** Peter A. Korevaar, C. Nadir Kaplan, Alison Grinthal, Reanne M. Rust, Joanna Aizenberg

**Affiliations:** 1 John A. Paulson School of Engineering and Applied Sciences, Harvard University, Cambridge, MA 02138 USA; 2 Institute for Molecules and Materials, Radboud University, Heyendaalseweg 135, 6525 AJ Nijmegen, The Netherlands; 3 Kavli Institute for Bionano Science and Technology, Harvard University, Cambridge, MA 02138 USA; 4 Department of Physics, Virginia Polytechnic Institute and State University, Blacksburg, VA 24061 USA; 5 Wyss Institute for Biologically Inspired Engineering, Harvard University, Cambridge, MA 02138 USA; 6 Department of Chemistry and Chemical Biology, Harvard University, Cambridge, MA 02138 USA

**Keywords:** Gels and hydrogels, Gels and hydrogels, Polymers

## Abstract

Materials that perform complex chemical signal processing are ubiquitous in living systems. Their synthetic analogs would transform developments in biomedicine, catalysis, and many other areas. By drawing inspiration from biological signaling dynamics, we show how simple hydrogels have a previously untapped capacity for non-equilibrium chemical signal processing and integration. Using a common polyacrylic acid hydrogel, with divalent cations and acid as representative stimuli, we demonstrate the emergence of non-monotonic osmosis-driven spikes and waves of expansion/contraction, as well as traveling color waves. These distinct responses emerge from different combinations of rates and sequences of arriving stimuli. A non-equilibrium continuum theory we developed quantitatively captures the non-monotonic osmosis-driven deformation waves and determines the onset of their emergence in terms of the input parameters. These results suggest that simple hydrogels, already built into numerous systems, have a much larger sensing space than currently employed.

## Abstract (web summary)

The role of hydrogels in a wide range of applications stems from its stimuli-responsive properties, but coupling of separate stimuli in hydrogels is not often shown. Here the authors demonstrate the emergence of counterintuitive transient phenomena in a polyacrylic acid hydrogel caused by distinct responses to different combinations of rates and configurations of sequential stimuli.

## Introduction

Hydrogels play a central role in a wide range of applications^1–11^, from drug delivery^12^ to microsensors^13^ to smart optical^14^ and homeostatic^15^ materials. Much of the recent interest has focused on enabling hydrogels to deform rapidly in-phase with specific inputs from the environment, such as pH^13,14^, temperature^16,17^ or chemical concentration^18,19^. In living systems, however, chemical signal transduction—from self-organizing amoebas navigating in fields of chemoattractant waves^20^, to heartbeats adapting to ionic bursts and spikes^21^, to membranes^22^ and genetic material reconfiguring with changing metabolic states^23^—often involves coupling multiple chemical stimuli arriving at separate times and rates. This non-equilibrium integration is driven by materials that convert each incoming stimulus into a long-lived active chemical or mechanical response, often outlasting the duration of the stimulus and thereby enabling it to be coupled to a later one. We considered that even simple hydrogels intrinsically possess these same mechanistic elements. In this way, hydrogels may potentially act as complex chemical signal integrators and in turn exhibit a wide range of previously unexplored transient phenomena and sensing behaviors.

In current strategies, there is a tight, in-phase feedback between the hydrogel deformations, diffusion, and reversible chemical reactions, such as protonation/deprotonation^7,13,14^, oxidation/reduction^24^, or complexation/dissociation^10,18^. This means that as soon as the stimulus—e.g. protons, divalent ions or reagents—has been removed from the environment, the gel returns to its original state. Then, the gel’s response to a subsequent stimulus is a new, separate, independent event. However, we hypothesized that introducing species that complex to the gel with variable, rather than uniformly fast, association/dissociation rates would enable common hydrogels to act as couplers of different stimuli separated across time and space. In particular, a slow dissociation rate should alter the traditional picture: By remaining complexed to the gel, a chemical stimulus would create a kinetically stable state with a characteristic lifetime. In such a case, the gel’s deformation would be transiently maintained upon removal of the stimulus from the environment. A second chemical species introduced later could then compete for binding sites, and trigger decomplexation of the first chemical species. As a result, the complexation, diffusion, and gel deformation rates associated with the first stimulus become interlinked with those of the second. In this paper, we show how coupling the dynamics of otherwise separate stimuli in time and space creates specific responses arising from the transient superposition of chemical species entering and exiting the gel.

**Fig. 1:**
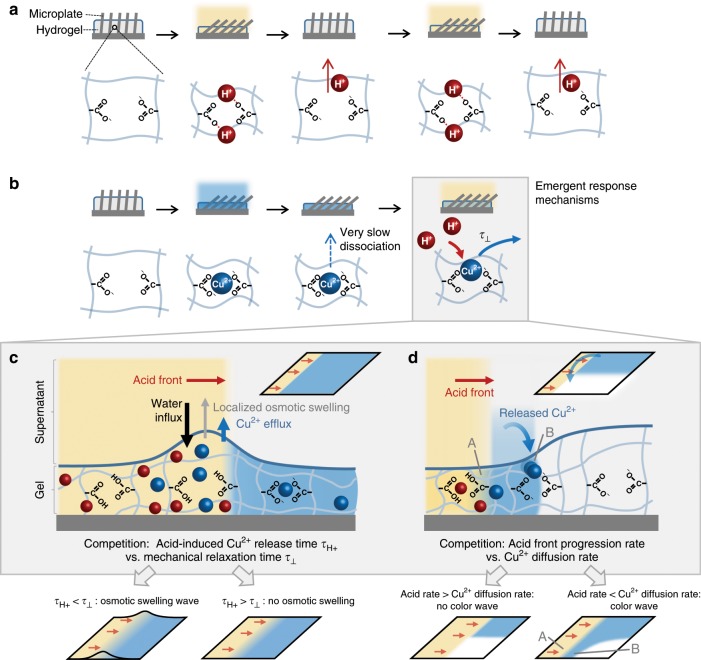
Non-equilibrium coupling of stimuli across time. **a** Traditionally, a responsive polyacrylic acid (PAA) hydrogel contracts and swells directly in-phase with the presence or absence of an acid stimulus (yellow). Here, hydrogel contraction tilts an array of embedded microplates (gray). **b** In contrast to this rapid reversibility, divalent cations (Cu^2+^, blue) contract the PAA gel by forming a kinetically stable complex with two carboxylate (COO^−^) groups, remaining in the gel after removal of Cu^2+^ from the environment. A subsequent acid stimulus then competes for COO^−^ groups and triggers dissociation of the Cu^2+^ on a timescale determined by its delivery rate (*τ*_H+_). The ensuing dynamics of diffusion, complexation, and mechanical deformations in the presence of the entering and exiting stimuli can lead to scenarios depicted in c-d: **c** Competition between transient water influx, induced by released Cu^2+^, and the mechanical relaxation time of the gel (*τ*_⊥_) creates traveling osmotic swelling waves reporting the speed of an oncoming acid front when *τ*_H+_ < *τ*_⊥_; **d** Competition between the diffusion and transient recomplexation of released Cu^2+^ (top, curved blue arrow) and its re-release by oncoming acid creates rate-sensitive traveling color waves when the acid progression rate is smaller than the Cu^2+^ diffusion rate (bottom right, narrow blue band).

We explore this concept with a widely used hydrogel, polyacrylic acid (PAA). Our system consists of a thin layer of hydrogel containing an array of embedded microplates, which enable real-time visualization of the gel’s deformations at the microscale. The hybrid hydrogel-microplate configuration^25^ has previously enabled a class of adaptive materials that catch and release biomolecules^26^, switch chemical reactions on and off^27^, or control wettability^28^, homeostasis^15^ and flow^29^. Under neutral or basic conditions, the carboxyl groups (COOH) of the PAA gel exist in a deprotonated form (COO^−^), the gel is swelled, and the embedded microplates stand upright. Consistent with the traditional use of PAA gel as a direct pH sensor, exposure to acid protonates the COO^−^ groups, inducing nearly immediate contraction of the gel and the associated tilting of the microplates (Fig. 1a, yellow). Adding a base rapidly deprotonates the gel and restores the original state. To test our hypothesis, we apply as a first stimulus divalent copper ions (Cu^2+^), which interacts with COO^−^ and contracts the gel. Cu^2+^ and COO^−^ form a kinetically stable chelate complex, which has been reported to maintain localized gel deformation and blue color over months in the absence of external Cu^2+^ (Fig. 1b, blue).^30^ Our results demonstrate how this blue color, characteristic for COO^−^-Cu^2+^-COO^−^ complexation, provides a complementary readout mechanism for the complex kinetic interplay between two stimuli. When acid (H^+^) is delivered as a second stimulus to a system previously exposed to Cu^2+^, H^+^ competes for COO^−^ groups (Fig. 1b, gray box) and displaces Cu^2+^, releasing it into the fluid phase of the gel and then into the initially copper-free supernatant. Cu^2+^ decomplexation will be dependent on the timescale of acid delivery *τ*_H+_. Varying *τ*_H+_ with respect to the timescales of Cu^2+^ diffusion and hydrogel deformation leads to the emergence of a variety of competing non-equilibrium dynamics (Fig. 1, expanded gray box).

Through experiments, scaling laws and a non-equilibrium continuum theory that captures the time-dependent coupling of the two stimuli, we demonstrate how two different, previously unseen responses emerge. (i) Acid-induced Cu^2+^ decomplexation inside the gel triggers transient water influx, driven by the osmosis caused by the Cu^2+^ ions released into the fluid phase of the gel (dependent on the timescale of acid delivery *τ*_H+_). At the same time, acid itself contracts the gel (with the mechanical relaxation time *τ*_⊥_). Counterintuitively, even though both Cu^2+^ and H^+^ contract the gel upon complexation, the competition between Cu^2+^-induced osmosis and acid-induced contraction produces traveling osmotic swelling waves when *τ*_H+_ < *τ*_⊥_ (Fig. 1c). (ii) If copper is complexed locally in the hydrogel, acid releases Cu^2+^ in region A to diffuse and recomplex to new COO^−^ groups in previously unoccupied neighboring regions B (Fig. 1d). At the same time, acid also competes with Cu^2+^ and displaces it from these new sites. As a result, traveling color waves appear ahead of a slow-moving acid front when it progresses more slowly than Cu^2+^ diffusion.

## Results

### Delivering the Cu^2+^ stimulus to the hydrogel-microplate system

**Fig. 2:**
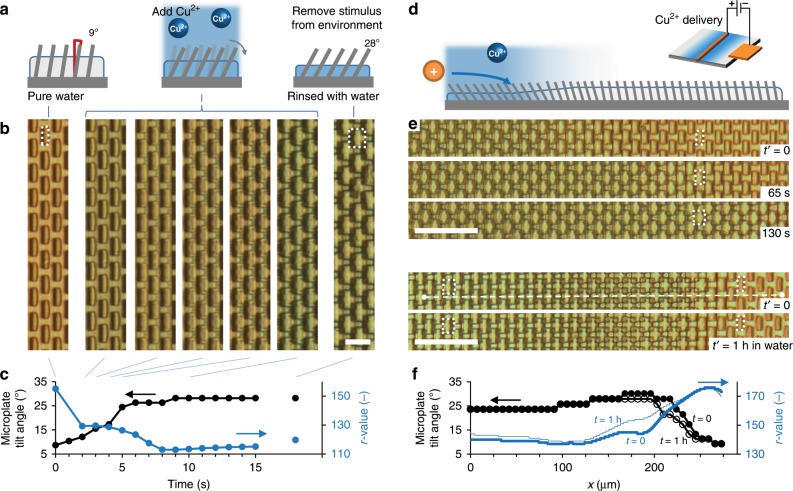
Delivery and storage of a Cu^2+^ stimulus. **a** Scheme of the Cu^2+^ complexation, hydrogel contraction, and microplate tilting upon exposure to Cu^2+^, and the maintenance of this response upon the formation of kinetically stable complexes after the stimulus is removed from the external environment. **b** Optical microscopy images showing that the addition of copper(II) sulfate (see Methods) leads to progressive microplate tilting, concurrent with a progressive colorless-to-blue transition of the hydrogel, indicative of COO^−^-Cu^2+^-COO^−^ complexation. The white dotted outlines indicate the change of the cross-sectional view of a single plate from rectangular (in the upright state) to nearly square (in the tilted state). Scale bar: 15 μm. **c** Data corresponding to microscopy images of the microplate tilt angle (black, reported as the angle between microplate and normal to the substrate, see Methods), and Cu^2+^ complexation (blue, reported as *r* value, i.e. red-channel value of the optical micrographs). The tilt angles and blue color are maintained after rinsing the substrate with water (right image in b). **d** Scheme showing Cu^2+^ ions electrochemically delivered from a positively charged copper electrode wire. **e** Upon applying a voltage of approx. 1 V across a copper wire (diameter approx. 100 μm), Cu^2+^ ions are released from the electrode (from the left side of the images), diffuse from left to right, and undergo complexation by the COO^−^ groups in the hydrogel, inducing blue color and microplate tilting. Scale bar: 50 μm. **f** After electrochemical delivery, localized storage of Cu^2+^ remains intact, with only a slow release of Cu^2+^ at the boundary of the contracted region. The *r* value and the microplate tilt angle vs. position *x*, shown in the graph, were acquired along the horizontal white dashed line shown in e. Scale bar: 50 μm.

Our hydrogel system comprises an array of surface-attached, slightly pretilted epoxy microplates embedded in a PAA hydrogel (Fig. 2a). The plates are 18 µm tall. The hydrogel has a height of *H* ≈ 10 µm measured from the confocal microscopy *z*-stack imaging (Supplementary Fig. 1). After deprotonating the PAA hydrogel by rinsing with a base, the hydrogel is swollen and the microplates are oriented nearly upright, 9° with the surface normal (see Methods for details). Upon addition of an aqueous copper(II)sulfate solution (0.8 M CuSO_4_), the hydrogel turns blue, indicating the formation of COO^−^-Cu^2+^-COO^−^ complexes in the hydrogel (Fig. 2b, c). Concurrently, the hydrogel contracts, and the embedded microplates tilt toward the substrate. This is evidenced by a progressive conversion from a rectangular to a square projection of the microplates in plain-view optical microscopy images. We note that the presence of the microplates and the blue color of the gel provide simple visual reporters on, respectively, (i) the deformation state of the gel, which is quantified by the microplate tilt angle, and (ii) Cu^2+^ complexation, which is quantified by the red channel (*r*-) value in optical microscopy images (see Methods and Supplementary Fig. 1). Both the microplate tilting and the blue color are maintained after Cu^2+^ is removed from the external solution, even after repeated rinsing with water, indicating a kinetically stable state that stores the Cu^2+^ stimulus upon complexation. The vertical diffusion of Cu^2+^ into the gel layer happens at a timescale *τ*_Cu2+_ ≡ *H*^2^/*D*_Cu2+_ ≈ 10 s, with a diffusion constant of *D*_Cu2+_ ≈ 10^−11^ m^2^ s^−1^. Thus, we expect the local contraction and coloring responses upon Cu^2+^ delivery to occur over a time *τ*_Cu2+_.

The Cu^2+^ delivery can be localized and made directional by using a thin copper electrode wire (diameter approx. 100 µm) mounted directly on top of the substrate, covered with a thin layer of a sodium perchlorate electrolyte solution (NaClO_4_, 0.05 M, see Scheme in Fig. 2d, Methods and Supplementary Fig. 2). When a voltage of approx. 1 V (current 0.1 mA) is applied, the microplates near the positive electrode begin to tilt as the corresponding region of the hydrogel contracts and turns blue. The region expands outward in time with a gradient of tilt angles and color intensity, consistent with Cu^2+^ ions diffusing from the electrode through the electrolyte and binding to the hydrogel (Fig. 2e and Supplementary Movie 1). The slight initial pretilting of the microplates in one orientation results in a uniform tilting direction upon Cu^2+^-complexation. As we noticed a variability in the degree of gel contraction depending on the direction of electrochemical Cu^2+^ delivery, all experiments were performed such that the pretilted plates were oriented towards the Cu^2+^ source, as schematically represented in Fig. 2d. Both the tilted state and blue color are maintained after Cu^2+^ is removed from the external solution by rinsing the substrate with water. Only a slow release of Cu^2+^ occurs at the edge of the Cu^2+^-contracted region (Fig. 2f).

### Osmotic pulses and waves selective to rapid Cu^2+^ release

**Fig. 3:**
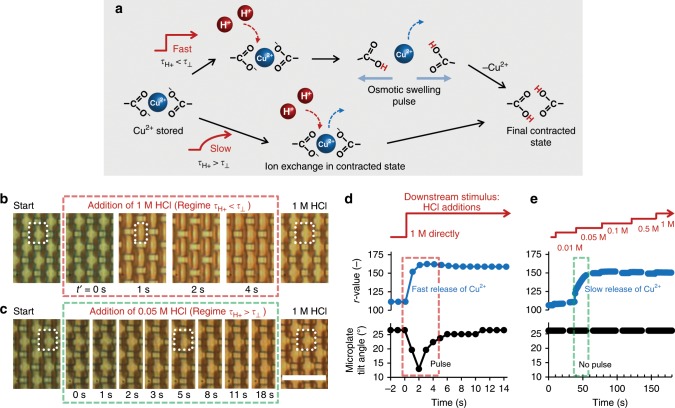
Cu^2+^ ions generate a transient osmotic swelling pulse upon rapid release by an acid stimulus. **a** Schematic presentation of the mechanism, showing how acid delivered after Cu^2+^ has been removed from the external environment of the hydrogel protonates carboxylate groups and thereby releases the complexed Cu^2+^. Fast release would generate an osmotic swelling pulse (top) before acid contracts the hydrogel again, while slow addition of acid should lead to a slow Cu^2+^ release without transient swelling (bottom). **b** Experimental demonstration of the fast Cu^2+^ release (regime *τ*_H+_ < *τ*_⊥_), triggered by direct addition of concentrated 1 M HCl, which results in rapid disappearance of the blue color and transient reorientation of the microplates to an upright position. The dotted outlines indicate the change of the cross-sectional view of a single plate from nearly square (in the tilted state) to rectangular (in the upright state), and back to nearly square. **c** Stepwise addition of acid leads to a slow release of Cu^2+^, such that the gel remains contracted without transient swelling (regime *τ*_H+_ > *τ*_⊥_). Scale bar: 25 μm. **d**, **e** Time-dependent microplate tilt angle and *r* value (acid stimulus added at *t* = 0) for fast (**d**) and stepwise, slow (**e**) addition.

The kinetically stable complexation creates a unique condition where Cu^2+^ is present inside the gel and absent from the external environment. Hence, rapid dissociation of Cu^2+^ upon protonation of the carboxylates must yield a transient osmotic pressure within the gel (Fig. 3a): If the release rate of Cu^2+^ is fast enough to induce water influx, this triggers an osmotic imbalance across the gel−supernatant solution interface. Satisfying this condition requires the relaxation time of the hydrogel deformation *τ*_⊥_ to be smaller than the diffusion timescale of Cu^2+^ (*τ*_Cu2+_), *i.e. τ*_⊥_ < *τ*_Cu2+_ ($\tau _ \bot \equiv {\it{\epsilon }}L/U^{(0)}$, where ${\it{\epsilon }}\sim {\mathrm{\Delta }}h/H$ is the ratio of the change in gel film thickness Δ*h* over its equilibrium thickness *H*, *L* is the horizontal length scale, and *U*^(0)^ is the inlet speed of the acid). Then, a sufficiently low acid-induced Cu^2+^ release timescale *τ*_H+_, such that *τ*_H+_ < *τ*_⊥_, is expected to produce an unusual transient gel swelling that would be selective only to fast onset-rates of the acid stimulus.

As an initial test of this scaling prediction, a concentrated acid solution (1 M HCl) was added to a hydrogel-microplate substrate containing complexed Cu^2+^. Directly after this delivery of a ‘fast arriving’ acid stimulus, a rapid dissociation of Cu^2+^ was observed, as indicated by the loss of blue color within *τ*_H+_ ≈ 2 s (Fig. 3b, d and Supplementary Movie 2). Concurrent with this color transition, the initially tilted microplates briefly stood upright at the onset of the acid stimulus, confirming that the system reports the fast acid flow with a transient swelling of the hydrogel when *τ*_H+_ < *τ*_⊥_, and then tilted back toward the substrate over *τ*_Cu2+_ ≈ 10 s. Corroborating that this unique transient swelling is indeed driven by an osmotic imbalance induced by Cu^2+^ dissociation, we show that the inclusion of CuSO_4_ (0.8 M) in the HCl solution—to reduce its hypotonic character—suppresses the swelling pulse (Supplementary Fig. 3).

To assess the selectivity of the swelling response for fast Cu^2+^ release, the same amount of acid was added slowly via a series of progressively concentrated HCl solutions, from 0.01 to 1 M. As shown in Fig. 3c, e, Cu^2+^ dissociates from the hydrogel during the addition step of 0.05 M HCl, over *τ*_H+_ ≈ 20 s. Since in this case the generation of free Cu^2+^ inside the gel is slower than its diffusion out of the gel (i.e. *τ*_H+_ > *τ*_Cu2+_ ≈ 10 s), the accumulation of free Cu^2+^ in the gel is insufficient to drive the osmotic swelling. As a result, the gel is observed to remain in its contracted state with the microstructures titled to the substrate, and simply changes color as protonation induces the release of Cu^2+^. We note that when calcium (Ca^2+^) is used as an alternative complexing agent to contract the PAA hydrogel, Ca^2+^ release upon rapid addition of acid induces a transient swelling response as well (Supplementary Fig. 4), suggesting a general applicability of our approach.

**Fig. 4:**
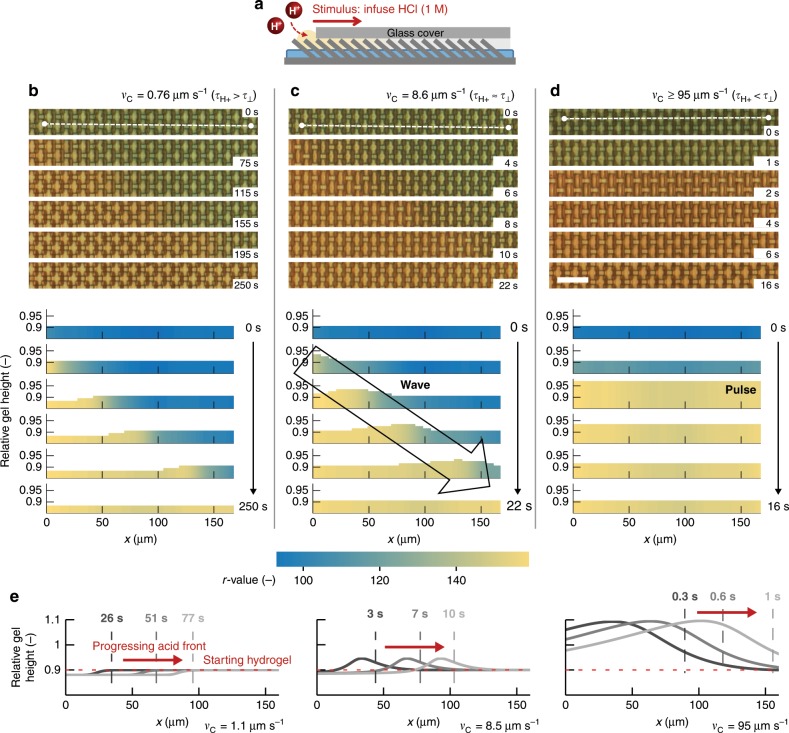
Traveling swelling waves that are sensitive to the acid progression rate. **a** Schematic of the experimental design: HCl (1 M) is added from the left side of a Cu^2+^-contracted substrate covered with a thin water film and a glass cover (see Methods). **b**–**d** (Top) Micrographs showing the progression of the acid stimulus at various rates, indicated by a blue-to-colorless transition. (Bottom) The height of the diagrams represents the evolution of the relative hydrogel height in time and space derived from the microplate tilt angle as described in Methods (Supplementary Fig. 1), along the white dashed line for the six micrographs from top to bottom; and the color of the diagrams represents the Cu^2+^ release as characterized by the blue-to-colorless transition: **b** No swelling pulse is observed for the acid stimulus that travels from left to right over 190 μm in 250 s (*v*_C_ = 0.76 μm s^−1^); **c**, **d** Faster progression of the acid within 22 s (**c** *v*_C_ = 8.6 µm s^−1^) and within 2 s (**d** *v*_C_ ≥ 95 µm s^−1^) generates swelling/contraction waves that travel at the acid front. Scale bar: 25 µm. **e** The results of our continuum theory show that traveling swelling/contraction waves are only obtained at *v*_C_ ≥ 8.5 µm s^−1^ for this set of experimental parameters, in excellent agreement with the experimental data. The red dashed lines indicate the starting height of the Cu^2+^-storing hydrogel; the vertical lines indicate the position of the progressing HCl front at three different times; the curves show the corresponding relative hydrogel height along the horizontal position *x*. The grayscale corresponds to three different times given in the legend of each plot.

The transient osmotic pressure due to rapid Cu^2+^ dissociation can also take the form of traveling swelling waves that are sensitive to the progression rate and direction of an acid front spreading across the substrate. As schematically shown in Fig. 4a, an acid stimulus with a controllable progression rate can be initiated by delivering a drop of acid under one edge of a glass cover (Methods and Supplementary Fig. 5). Cu^2+^ decomplexation at the acid front is indicated by a blue-to-colorless transition that progresses from left to right (Fig. 4b, c), and occurs over a length scale of *L* ≈ 100 µm, consistent with free diffusion within the stimulus front (*D* ≈ 10^−9^ m^2^ s^−1^) over *τ*_Cu2+_ ≈ 10 s and *τ*_⊥_ ≈ 10 s (see Supplementary Information). To meet the condition of *τ*_H+_ < *τ*_⊥_ ≈ 10 s—the requirement for observing a transient swelling response as discussed above, where *τ*_H+_≡ *L/v*_C_—the acid progression speed must be *v*_C_ *>* 10 µm s^−1^. Consistent with this prediction, a wave of weakly up-and-down moving microplates is experimentally observed to travel at the front of an acid stimulus moving with a minimum rate of *v*_C_ = 8.6 µm s^−1^ (Fig. 4c and Supplementary Movie 3). A slower progression yields no swelling pulse at the stimulus front (Supplementary Fig. 6), as exemplified by the results in Fig. 4b acquired at *v*_C_ = 0.76 µm s^−1^. In contrast, fast progression (*v*_C_ ≥ 95 µm s^−1^) yields a high-amplitude traveling pulse (Fig. 4d). The pressure that is required to establish a swelling wave spreading over *L* ≈ 100 µm within *τ*_H+_ ≈ 10 s determines the poroelastic diffusion constant of water inside the hydrogel, given by *D*_water_ ≡ *k*_f_*∙p̅*/*μ*_f_ ≈ 10^−10^ m^2^ s^−1^, where *k*_f_ ≈ 10^−19^ − 10^−18^ m^2^ is the hydraulic permeability of the hydrogel and *μ*_f_ = 10^−3^ Pa s is the dynamic viscosity of water. The required pressure $\bar p$ equals 1−10 MPa; a pressure that can be generated upon osmosis as the concentration of Cu^2+^ ions is estimated to be 2.9 M (Supplementary Fig. 7 and Supplementary Information), implying a maximum osmotic pressure of ~7 MPa (*p*_osm_ ≈ [Cu^2+^]∙k_B_*T*). We note that the orientation of the microplates with respect to the acid stimulus progression does not have a major effect on the swelling response of the hydrogel.

To further assess the timescales and forces involved in the unique transient swelling responses and traveling waves that arise upon coupling of successive Cu^2+^ and acid stimuli, we developed a continuum theory that gives the time-dependent height profile of a thin hydrogel sheet, based on time- and position-dependent descriptions of (i) Cu^2+^ and acid present in the supernatant fluid, in the hydrogel interior fluid, and complexed to PAA; (ii) the osmotic and contractile forces exerted on the gel due to free and complexed Cu^2+^ and acid in the gel, and (iii) the mechanical deformation of the gel (see Supplementary Discussion). Simulations based on parameter values, which match experimentally assessed time- and pressure-scales, quantitatively reproduce the experimental vertical deformation waves of the hydrogel, as derived from the experimentally observed microplate tilting waves (Fig. 4e, Supplementary Figs. 8 and 9, and Supplementary Movies 4–6). The transient osmotic vertical flow for thin film domains is given by Supplementary Eq. 14 and holds at the leading order O(*δ*^0^, *ϵ*^0^), where *δ* is the aspect ratio of the thin film; both *ϵ* and *δ* are very small. The mobility coefficient in Supplementary Eq. 14 scales with *δ*^−2^ and is not a free parameter. This osmotic flow term quantitatively reproduces the osmosis-induced traveling waves (Fig. 4, Supplementary Movies 4–6). Thereby, our theory shows that, first, species released within the hydrogel induce transient osmosis; second, this enables unique signaling routines that selectively report input stimuli occurring at fast rates; and, third, swelling pulses are displayed at timescales that cannot be established by solely breaking crosslinks in the hydrogel.

### Traveling color waves reporting slow acid fronts

Copper ions released by acid from the hydrogel into an otherwise Cu^2+^-free medium not only enable short-term osmotic pressure in the gel, but also give rise to localized patterns of recomplexation as the released Cu^2+^ ions diffuse with the moving acid front. While swelling waves require a rapidly moving acid front to trigger a rapid release of Cu^2+^ inside the gel, recomplexation of Cu^2+^ should in contrast require the acid front to be moving slowly enough for the diffusing Cu^2+^ ions to be able to compete with the oncoming protons for new binding sites. Assuming a graded acid concentration at the front, Cu^2+^ comigrating with the front will potentially have a time window to recomplex to the gel in the presence of a low acid concentration, before saturating acid overtakes the recomplexed Cu^2+^ and releases it again. Consistent with this possibility, flowing a solution containing 1 M HCl and 0.8 M CuSO_4_ with a slow progression rate along a substrate with a deprotonated PAA hydrogel yields a transient band of Cu^2+^ complexation at the solution front (*v*_C_ = 3 µm s^−1^, Supplementary Fig. 10). For a system that is exposed first to Cu^2+^ and subsequently to progressing acid, initial release of Cu^2+^ by acid at a region A, followed by diffusion of Cu^2+^ through the supernatant solution and recomplexation to the gel at a region B, can result in a transient band of Cu^2+^ complexation. This must only happen when acid migration from A to B is slower than the diffusion of Cu^2+^ and its subsequent recomplexation at B: *L*_*x*_/*v*_C_ > *L*_*x*_^2^/*D*^(a)^ + *τ*_Cu2+_, where *L*_*x*_ ~ *L* ≈ 100 μm is the distance between A and B, and *D*^(a)^ is the diffusivity in the supernatant solution.

**Fig. 5:**
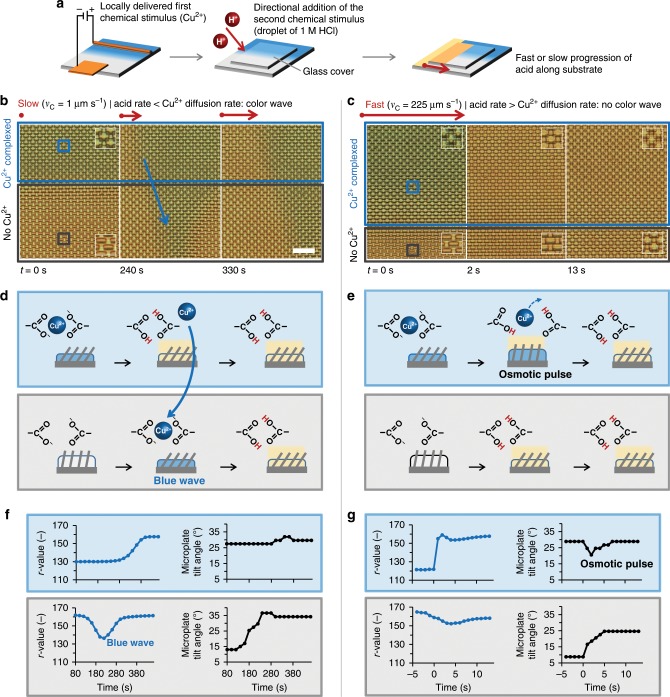
Traveling color waves selectively reporting slowly progressing acid stimuli. **a** Schematic presentation showing the mechanism for the appearance of the travelling color waves: Cu^2+^ is initially delivered electrochemically to one side of the substrate (blue region in diagram). A glass cover is then applied and the acid is added from the left and allowed to progress along the substrate. **b** Experimental demonstration of the slow acid progression: Cu^2+^ is complexed in the region of the substrate where it was applied; slow progression of the acid (acid rate < Cu^2+^ diffusion) allows Cu^2+^, released at the acid front in the Cu^2+^-complexing region (blue box), to migrate to the adjacent Cu^2+^-free region (blue arrow), generating a transient blue wave just ahead of the stimulus front (gray box). Scale bar: 50 µm. **c** Fast progression of the acid from left to right (acid rate > Cu^2+^ diffusion) induces a swelling/contraction wave in the Cu^2+^-complexing region (blue box) and a direct contraction of the hydrogel with no color wave in the region with no Cu^2+^ (gray box). **d**, **e** Schematic representation of the subsequent stages for both regions in (**b**) and (**c**), respectively. **f**, **g** Time-dependent *r* value and microplate tilt angle, acquired at the blue and gray squares in (**b**) and (**c**), respectively.

To test this idea, we electrochemically delivered Cu^2+^ across one half of a gel/microplate system, so that Cu^2+^ is stored on one side while the other remains copper-free (blue and white sides in Fig. 5a, respectively). Acid is then flowed such that the front progresses over both halves in parallel (Fig. 5a, yellow). This configuration potentially allows some of the released Cu^2+^ at the front to diffuse to and recomplex on the copper-free side, subject to the acid-dependent competition and time window. Our experimental results at different progression speeds indeed indicate the ability of this mechanism to produce a distinctive slow-rate-sensitive response: a slow acid progression speed (*v*_C_ = 1 µm s^−1^) generates a wave of blue color that travels with the acid front through the initially copper-free side (Fig. 5b, d, f and Supplementary Movie 7), featuring the regime where the acid progression is slower than the Cu^2+^ diffusion. This response was further observed with an intermediate rate of *v*_C_ = 3 µm s^−1^ (Supplementary Fig. 11), and also with a hydrogel without embedded microplates (Supplementary Fig. 12). The inequality *L*_*x*_/*v*_C_ > *L*_*x*_^2^/*D*^(a)^ + *τ*_Cu2+_ only holds when *v*_C_ < 5 µm s^−1^, assuming *D*^(a)^ = 10^−9^ m^2^ s^−1^ and *τ*_Cu2+_ = 10 s. Indeed, at fast progression rates (*v*_C_ = 225 µm s^−1^, Fig. 5c, e, g), no blue color was observed on the copper-free side (acid progression rate > Cu^2+^ diffusion rate). Instead, a wave of osmotic pressure was generated on the copper-storing side, with the associated transient upright movement of the microplates, as discussed above.

## Discussion

Our results provide a potentially transformative approach to chemical signal processing and, more generally, suggest that simple hydrogels have a much larger sensing space than is currently made use of. By integrating the complexation-transport-deformation dynamics induced in the gel by two chemical stimuli that occur separately in time and space, we show that a common hydrogel—traditionally used for direct stimulus tracking through nearly in-phase response to an applied stimulus—can produce previously unseen complexity. This is demonstrated by time-sensitive, nonmonotonic osmotic effects accompanied by spikes and waves of gel expansion and contraction, as well as traveling color waves of patterned migration and recomplexation. Our non-equilibrium continuum theory captures how the diverse responses depend on the coupling of diffusion, flow, complexation, and hydrogel deformation as successive chemical stimuli enter and exit the gel. The theory allows the parameter windows to be predicted for a range of phenomena based on the relative timescales involved in signal coupling. Combined with an extensive experimental and scaling analysis, the model provides insight into the competing processes underlying the response mechanisms and emergent behaviors. As exemplary cases, our theory reveals how traveling osmotic swelling waves can emerge in response to the rapid onset of a stimulus that would normally—on its own—contract the gel, if the timescale of acid propagation is smaller than the mechanical relaxation time of the hydrogel. The theory further implies that, when two H^+^ signals approach from opposite directions, the accompanying swelling fronts would annihilate each other upon collision. This is because no available bidendate complexation sites would be left ahead of each front to be decomplexed and create osmotic imbalance. Further scaling laws elucidate how a slowly moving acid gradient can induce sequences of migration and recomplexation highly sensitive to the interdependent dynamics of the released and oncoming stimuli.

In conclusion, the framework presented here shows how a hydrogel can be used without specialized modifications to perform complex chemical sensing tasks not previously achieved with electronics-free systems. The exemplary responses we demonstrate likely represent only a small sample of the dynamic phenomena that may emerge. Based on simple, reversible chemistry and trivial hydrogel composition and geometry, our scaling analyses and the theoretical model elucidate distinct outputs able to discriminate among many possible combinations and permutations of rates, times, sequences, and configurations of multiple arriving stimuli. These concepts are potentially applicable to a wide range of hydrogels, stimuli and non-equilibrium molecular systems beyond the ions, acid, and PAA gel used in this study.^31–39^ The non-equilibrium concepts and theory can be further applied to readout mechanisms beyond the microplates used in this study, such as via microparticles dispersed within the gel or via focussing and defocussing of light beams by the gel. Additionally, the concept of rate-selective recomplexation waves—exemplified by the blue color waves in our system—can be expanded by selecting alternative pairs of complexing agents (such as Ca^2+^ and H^+^), potentially in combination with fluorescent or other indicators. In particular, the non-equilibrium mechanisms revealed in this study may enable micron-scale synthetic soft actuators, analogous to the way Ca^2+^-based biochemical reaction-transport pathways power the motion of some single-celled organisms, such as *D. discoideum*^40^ and the *Vorticella* ciliates^41^. Beyond reporting the gel’s dynamics, microstructures embedded in the gel can themselves introduce feedback to the complexation-transport-deformation coupling^42^, potentially opening another realm of non-equilibrium sensing. Further developing these capacities may bring about new possibilities for integrating complex chemical sensing and transduction, using simple soft materials, into areas such as soft robotics, catalytic materials, and agricultural and biomedical diagnostics.

## Methods

### Chemicals and materials

Polydimethylsiloxane (PDMS, Dow-Sylgard 184) was purchased from Dow Corning Corporation (Midland, MI, USA). Epoxy resin OG178 was purchased from Epoxy Technology (Billerica, MA, USA). Glycidyl methacrylate, acrylic acid, sodium acrylate, 2,2′-azobis(2-methylpropionamidine) dihydrochloride, *N*-*N*′-methylenebisacrylamide, 1-butanol, ethylene glycol, copper(II)sulfate, sodium perchlorate, ethylenediaminetetraacetic acid, potassium hydroxide and hydrochloric acid were purchased from Sigma Aldrich. Irgacure 819 was purchased from BASF Corporation, Lumiprobe BDP FL NHS ester from Lumiprobe Corporation (Hallandale Beach, FL, USA), calcium chloride from J.T. Baker and copper(II)chloride from Fluorochem. All compounds and materials were used as received.

### Fabrication of hydrogel-embedded microplate substrates

To prepare the epoxy microplate substrates, first a PDMS negative mold was obtained by curing a 10:1 wt./wt. mixture of base resin and hardener onto a silicon master with the microplates positioned in a staggered array, with a height of 18 µm, a width of 10 µm, a thickness of 2 µm and a spacing of 5 µm in both *x* and *y* directions. The silicon master was fabricated via the Bosch process and functionalized with (tridecafluoro-1,1,2,2-tetrahydrooctyl)trichlorosilane in a desiccator under vacuum at room temperature for at least 24 h, in order to facilitate demolding of the PDMS. The PDMS prepolymer mixture was mixed for 1 min, degassed under vacuum at room temperature, poured over the silicon master in a petri dish, put under vacuum at room temperature to remove bubbles, and then cured at 70 °C. After 2 h, the PDMS molds were cooled and peeled off from the silicon mold. To prepare an epoxy microplate substrate, 35 µL of a 9:1 (wt./wt.) prepolymer mixture of the OG178 epoxy resin and glycidyl methacrylate was added to the PDMS mold and covered with a glass slide (16 × 16 mm^2^, pretreated in O_2_-plasma for 2 min). UV curing was performed under a UV lamp (100 W, Blak-Ray with a 365 nm band-pass filter, approx. 10 mW cm^−2^ at 365 nm) for 30 min. The microplate substrate was then obtained by carefully removing the glass slide from the PDMS mold.

In order to embed the microplate structures in the hydrogel, 3 µL of a hydrogel precursor solution was added to the substrate. The hydrogel precursor solution was prepared by combining 400 µL of acrylic acid with 20 mg *N*-*N*′-methylenebisacrylamide crosslinker in 1 mL of a 1:1 v/v mixture of ethylene glycol and 1-butanol. To introduce the Irgacure 819 photoinitiator, 10 µL of a 25 mg mL^−1^ solution in 1-butanol was added to 90 µL of the aforementioned solution to obtain the hydrogel precursor solution. After applying the hydrogel precursor solution to the microplate substrate, it was immediately covered with a thin glass cover slide (cleaned with isopropanol) and the hydrogel was subsequently cured for 5 min under UV, similarly to the epoxy curing. After curing, the hydrogel-microplate substrate was immersed in deionized water to allow the glass cover slide to detach and to exchange the ethylene glycol/1-butanol mixture in the hydrogel for water.

To assess the embedding of the microplates in the hydrogel, the hydrogel was dyed by combining a solution of Lumiprobe BDP FL NHS ester (2.5 mg mL^−1^) in a 1:1 v/v 1-butanol/ethylene glycol mixture with an equal volume of a double concentrated hydrogel precursor solution (see above). Next, the obtained solution was applied to the microstructures and cured as described above. The dyed hydrogel-microplate substrates were then analyzed by confocal microscopy (*λ*_ex_ = 488 nm).

To prepare a hydrogel substrate with no microplates embedded, first an epoxy substrate was prepared by photo curing a Norland 68 epoxy resin sandwiched between a flat PDMS support layer and a glass cover (prepared as described above, total exposure time under UV 10 min). Subsequently, 40 µL of a hydrogel precursor (113 mg mL^−1^ sodium acrylate, 11 mg mL^−1^ *N*-*N*′-methylenebisacrylamide and 7.5 mg mL^−1^ 2,2′-azobis(2-methylpropionamidine) dihydrochloride photoinitiator in water) was applied, and covered with a glass slide of 18 × 18 mm^2^. Subsequently, the hydrogel was cured under UV (366 nm, 4 min) and the substrate was immersed in water to detach the glass cover. Then, the substrate was vertically immersed for 2 min in an aqueous CuCl_2_ (0.8 M) solution, such that one half of the hydrogel was complexed to Cu^2+^ as evidenced by the appearance of blue color. The results in Supplementary Fig. 12 were acquired in analogy to the methodology applied for Fig. 5; the images were acquired on a Leica DM 2500 microscope equipped with a Leica DFC 7000T camera.

### Assessing complexation of Cu^2+^ and tilting of microplates

All optical microscopy images were acquired with an Olympus IX71 dark field inverted microscope equipped with a QImaging Retiga 2000R camera unless stated otherwise. All colored images were acquired with similar white balance settings and light intensity. Confocal microscopy was performed using a ZEISS LSM 700 microscope. SEM images were acquired on a JEOL JSM 639OLV scanning electron microscope, and the sample was sputter-coated with Au/Pd for imaging.

To quantify the tilting of the microplates, the microplate tilt angle was determined from the microplates’ projection in optical microscopy images. The projection of the microplates was measured in the images and, based on the ratio of this projection to the distance between *n* rows of microplates in the same image, which equals (*n* *–* 1) × 7 μm, converted to the real dimensions *p* in μm. Based on the height of the microplates *h* = 18 μm and the thickness *t* = 2 μm, the microplate tilt angle *α* was determined as *α* = 90− acos((*p* − *t*)/*h*) (see Supplementary Fig. 1c). It is assumed that the plates do not curve upon actuation but maintain their straight form and only hinge at the connection to the substrate (see Supplementary Fig. 1b). The relative gel height was derived via cos(*α*)/cos(*α*_gel completely swelled_).

The color profiles were acquired using ImageJ 1.50b software. To avoid the profiles being disturbed by the contours of the microplates, the images were blurred (Gaussian blur; Sigma radius 50) prior to acquiring the *r* value (red channel RGB value).

Absorption spectra were acquired on a Beckman Coulter DU 720 UV/Vis spectrometer, in a polymethyl methacrylate (PMMA) cuvette (optical path length 1 cm) at room temperature, and the background was acquired on a PMMA cuvette with water.

### Complexation of Cu^2+^ in the hydrogel

Prior to the contraction of the hydrogel via Cu^2+^ complexation, the hydrogel-microplate substrate was sequentially rinsed with hydrochloric acid (HCl 1 M, 4× the same solution of 2 mL), water (5×), potassium hydroxide (KOH in a concentration of 0.1 M, 4× the same solution of 2 mL, repeated with a fresh solution of 2 mL), and water (5×). Thereafter, excess water was removed from the substrate with a tissue. For Fig. 2b, a thin layer of 50 µL water was applied to the substrate, and subsequently 10 µL CuSO_4_ 0.8 M was added. To assess the storage of Cu^2+^ upon complexation to the hydrogel, the substrate was rinsed with water (4×).

### Electrochemical delivery of Cu^2+^

Cu^2+^ ions were delivered to the hydrogel-microplate substrate by mounting a copper wire (diameter approx. 100 μm) as a positive electrode and a copper mesh (hole and wire diameter approx. 100 μm) as a negative electrode on top of the substrate with scotch tape, with a distance between the (+) and (–) electrodes of approx. 3 mm, as schematically represented in Fig. 2d. The scotch tape was applied such that it did not allow a short-circuit between the electrodes. One hundred microliters sodium perchlorate (NaClO_4_) in water (0.05 M) was added as an electrolyte solution, forming a thin electrolyte layer that ensured contact with both the (+) and (–) electrodes. The electrodes were connected via crocodile clips to a Keithley 2450 Sourcemeter power supply, and the current was set at 0.1 mA, resulting in a voltage of approx. 1 V.

### Swelling and contraction pulses

To prepare the hydrogel for Cu^2+^ complexation, the hydrogel was rinsed with hydrochloric acid (HCl 1 M, 4× the same solution of 2 mL), water (5×), potassium hydroxide (KOH 0.1 M, 4× the same solution of 2 mL, repeated with a fresh solution of 2 mL), and water (5×). Subsequently, excess water was removed from the substrate with a tissue, 50 µL of a 0.8 M CuSO_4_ solution was added, excess Cu^2+^ was removed by rinsing the substrate with water and excess water was removed with a tissue. To obtain the swelling/contraction pulse (Fig. 3b), 1 mL 1 M HCl was added. The stepwise addition of HCl solutions with increasing concentrations (Fig. 3c) was performed by adding volumes of 1 mL, with removal of excess HCl solution from the substrate prior to each subsequent addition.

### Controlled progression of acid stimulus

Cu^2+^ was first complexed to the hydrogel as described above (swelling and contraction pulses). The substrate was then dried with a tissue, 4 μL water was applied, and the substrate was covered with a 10 × 16 mm^2^ glass cover of 1 mm thickness. To initiate the HCl stimulus, a droplet of 30 µL 1 M HCl was added at the edge of the glass cover as schematically shown in Fig. 4a. The color transition progression speed *v*_C_ in µm s^−1^ was determined via the time it took the blue-to-colorless front to progress from left to right over the field of view (190 µm). Small-magnification optical microscopy images in Supplementary Fig. 5 reveal a fast progression of the HCl front over the first few millimeters, whereas further away from the edge of the glass cover the progression of the HCl front slows down, enabling variation of *v*_C_ for different experiments shown in Fig. 4 and Supplementary Fig. 6. Alternatively, a larger amount of water under the glass cover can be used to slow down the progression.

### Spatial patterning of pulses and traveling waves

To obtain a localized Cu^2+^ complexation (Fig. 5), Cu^2+^ was electrochemically delivered via the same procedure as described above (electrochemical delivery of Cu^2+^). Here, the experiments started with a substrate that was rinsed with hydrochloric acid (HCl 1 M, 4× the same solution of 2 mL), water (5×), potassium hydroxide (KOH 0.05 M, 4× the same solution of 2 mL, repeated with a fresh solution of 2 mL), and water (5×). Subsequently, the electrodes were removed, and the substrate was rinsed with water, dried with a tissue, and covered with 4 µL water and a glass cover (10 × 16 mm^2^, 1 mm thick). Similarly to the procedure described above (Controlled progression of acid stimulus), a droplet of 30 µL 1 M HCl was added at the edge of the glass cover to initiate the Cu^2+^ release, as schematically shown in Fig. 5a.

### Determining the concentration of Cu^2+^ complexed to gel

The concentration of Cu^2+^ complexed to the COO^−^ groups in the hydrogel was determined upon extraction of Cu^2+^ from the hydrogel with an ethylenediethylaminetetraacetate (EDTA) solution, as shown in Supplementary Fig. 7. By comparing the optical density of the extract solutions to a calibration line (based on absorption spectra of aqueous EDTA solutions (0.27 M, 1 M KOH) with different CuSO_4_ concentrations), the total amount of Cu^2+^ ions was determined. For the hydrogel-microplate substrate, we obtained a total Cu^2+^-amount of 0.0038 mmol. Based on the ratio between the area of the blue region in Supplementary Fig. 7b and the printed squares of the paper background (0.634 × 0.634 cm^2^), the hydrogel area in the sample is estimated to be 1.30 cm^2^. Based on the estimated thickness of the contracted hydrogel of 10 μm (Supplementary Fig. 1), the volume of the hydrogel is 0.00130 cm^3^. Therefore, the Cu^2+^ concentration inside the contracted hydrogel is estimated to be 0.0038 mmol/0.00130 cm^3^ = 2.9 M (Supplementary Fig. 7). The concentration of carboxylic acid groups in the hydrogel is estimated from the precursor solution, which was prepared from a solution of 0.4 mL acrylic acid + 0.5 mL ethylene glycol + 0.5 mL 1-butanol, and was subsequently mixed in a 9:1 ratio with the initiator solution, resulting in an acrylic acid concentration of 3.74 M. After the application of the hydrogel precursor, we assume that the solution wets the plates, with a height of 18 μm, as well as the glass cover applied on top of it. Densification of this precursor solution with a thickness of 18 μm to a hydrogel with a final thickness of 10 μm (see Supplementary Fig. 1) results in a final carboxylic acid concentration of 6.7 M. This indicates that after exposing to a concentrated CuSO_4_ solution, the Cu^2+^ to COO^−^ complexation in the hydrogel approaches a 1:2 ratio (Cu^2+^/COO^−^_max_ = 43%).

## Supplementary information

Supplementary Information

Description of Additional Supplementary Files

Supplementary Movie 1

Supplementary Movie 2

Supplementary Movie 3

Supplementary Movie 4

Supplementary Movie 5

Supplementary Movie 6

Supplementary Movie 7

## Data Availability

The data that support the findings of this study are available within the article (and its Supplementary Information files) and from the corresponding authors on reasonable request.

## Code availability

The computer code that was developed to perform the simulations with our model is freely available at Github: https://github.com/nadirkaplan/hydrogels_naturecomm.
