# Supplementary material for: Non-equilibrium signal integration in hydrogels
Source: Nat Commun. 2020 Jan 20;11:386. doi: 10.1038/s41467-019-14114-0 (PMC6971035; doi:10.1038/s41467-019-14114-0)
Supplement: Supplementary file 1 — Supplementary Information [file 41467_2019_14114_MOESM1_ESM.pdf]

Non-equilibrium signal integration in hydrogels

Korevaar et al.

Supplementary Figures

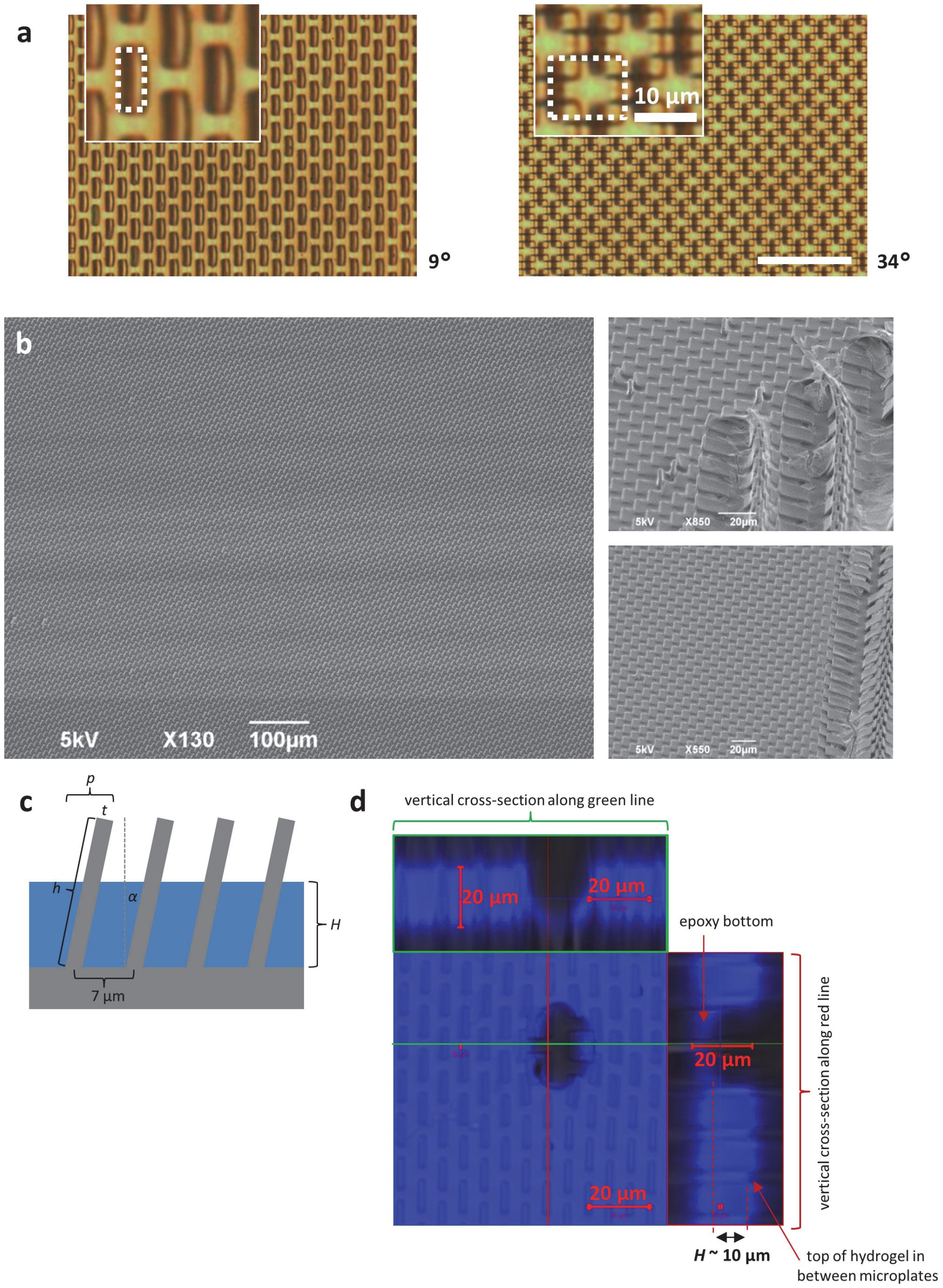

Supplementary Figure 1. Characterization of the geometry of the polyacrylic acid hydrogel system with embedded microplates. **a,** Optical microscopy images. Left: Under basic conditions (0.01 M potassium hydroxide, KOH) the hydrogel is swollen and the microplates are oriented upright. Right: Under acidic conditions (1 M hydrochloric acid, HCl) the hydrogel is contracted and microplates are tilted toward the substrate. The scale bar equals 50 µm. **b,** Scanning electron

microscopy (SEM) images acquired when the sample is tilted 0° (left), and 45° (right). The regions on the right were specifically chosen to present artefacts, which correspond to spots on the substrate where the hydrogel is missing, thus showing that the microplates do not curve significantly upon hydrogel contraction. This justifies deriving the tilt angle of the microplates from their top-view projection in optical microscopy images. **c,** Schematic diagram showing the correlation between the top-view projection and the tilt angles (see Methods). **d,** Z-stack confocal microscopy images of epoxy microplates embedded in a polyacrylic acid hydrogel that is dyed with Lumiprobe BDP FL NHS ester, acquired under swollen conditions. The hole in the middle of the image represents an artefact in the sample where no hydrogel is present. Based on the cross section of the hydrogel-microplate substrate, we estimate that the hydrogel has a height H of approx. 10 μm.

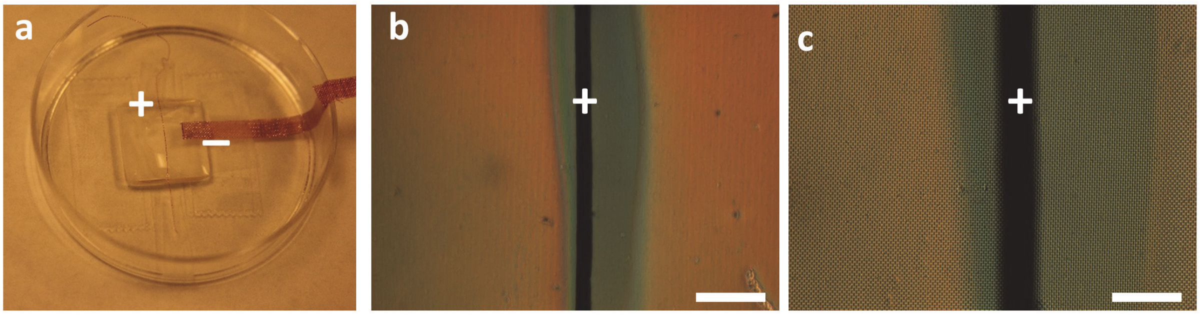

Supplementary Figure 2. a, Photograph of the setup used to deliver Cu^{2+} ions electrochemically to the hydrogel-microplate substrate, showing the hydrogel-microplate substrate (mounted in a petri dish), the (+) copper wire electrode, the (-) copper electrode mesh and 100 µL sodium perchlorate (0.05 M) solution that forms a thin film that wets the substrate as well as both electrodes. **b,c** Optical microscopy images acquired after delivering Cu^{2+}, showing a blue region indicative of Cu^{2+} complexation on both sides of the (+) electrode (black line in the middle). The scale bar in (b) equals 500 µm, the scale bar in (c) equals 200 µm.

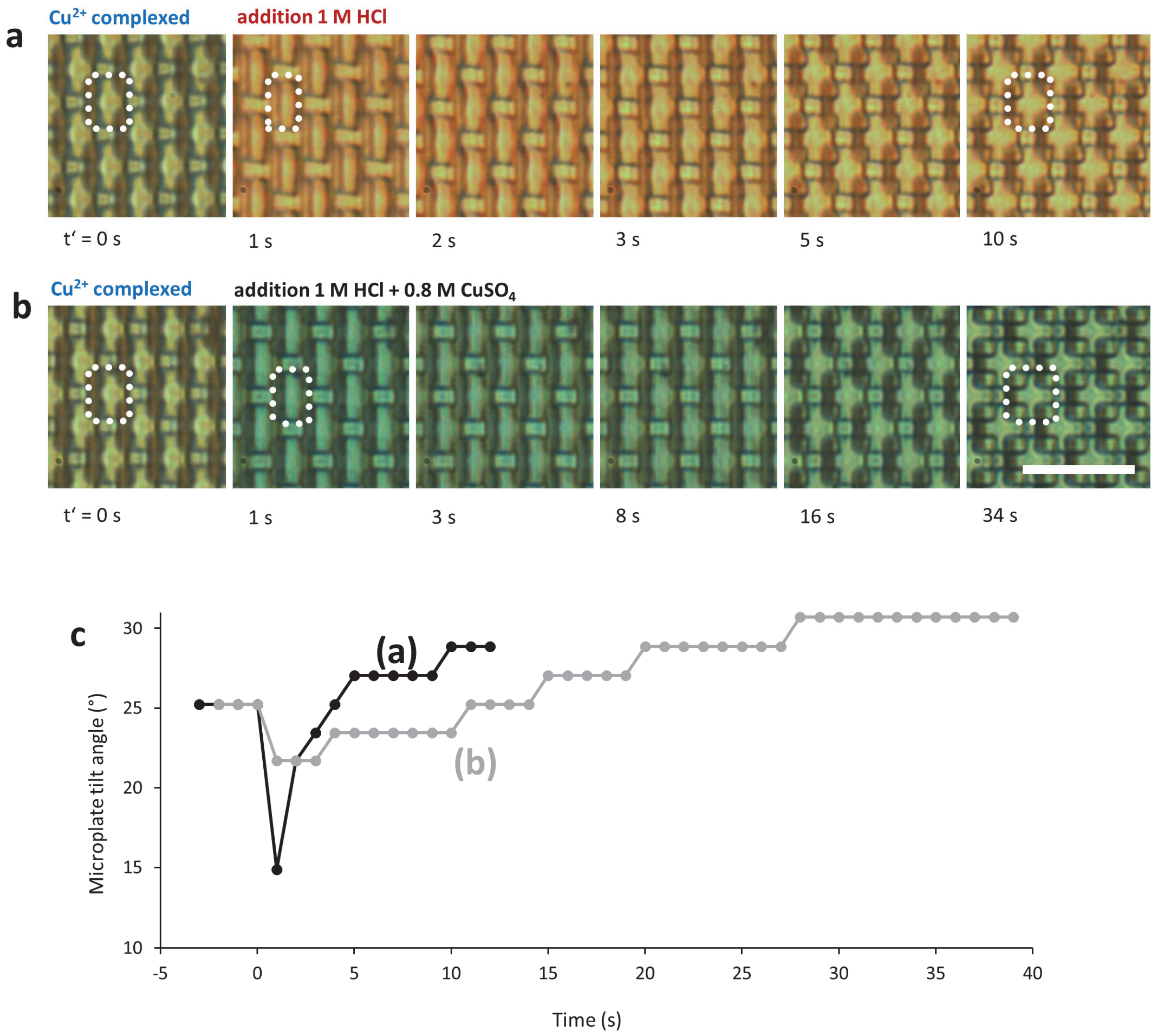

Supplementary Figure 3. a, Optical microscopy images acquired during the addition of 1 M HCl to a hydrogel-microplate substrate that was initially contracted upon Cu^{2+} complexation (upon addition of 0.8 M $CuSO_4$ in water to a substrate covered with a thin film of water). **b**, In analogy, a 1 M HCl solution containing 0.8 M $CuSO_4$ was added to a Cu^{2+}-contracted substrate. Here, a reduced pulse intensity is observed (*i.e.*, a lower decrease in tilt angle), suggesting that including $CuSO_4$ in the HCl solution reduces its hypotonic character, and therefore less osmotic swelling of the hydrogel is obtained. **c**, Microplate tilt angle *vs.* time, for the experiments shown in (a) and (b). Scale bar: 25 µm.

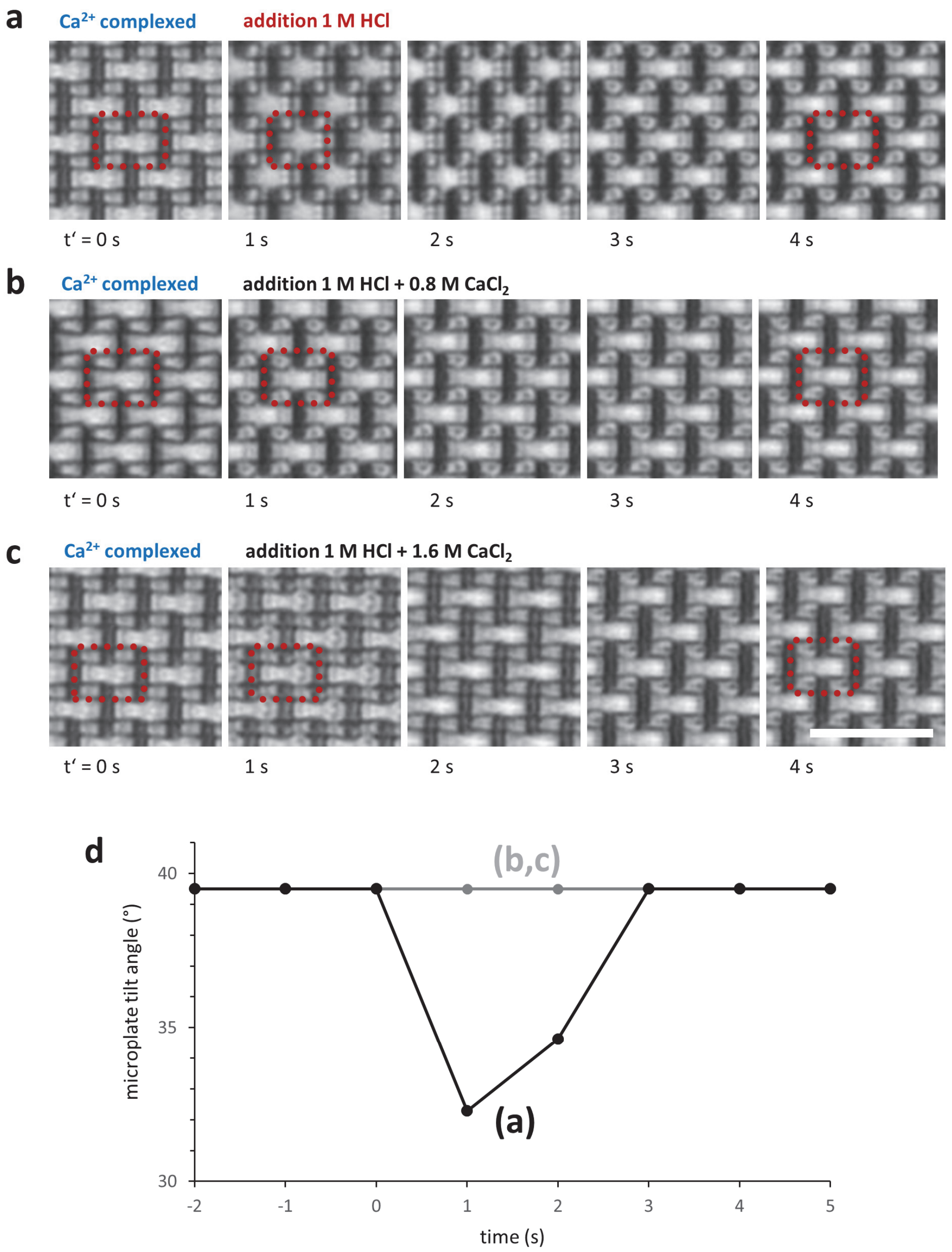

Supplementary Figure 4. a, Optical microscopy images acquired during the addition of 1 M HCl to a hydrogel-microplate substrate that was initially contracted upon Ca^{2+} complexation (upon the addition of 5 µL 0.8 M calcium chloride ($CaCl_2$) in water to a substrate covered with a thin film of water). **b, c,** In analogy to Supplementary Fig. 3, 1 M HCl solutions containing 0.8 M $CaCl_2$ (b) and 1.6 M $CaCl_2$ (c) were added to a Ca^{2+}-contracted substrate, resulting in no pulse. Scale bar: 25 µm. **d**, Microplate tilt angle *vs.* time, for the experiments shown in a-c.

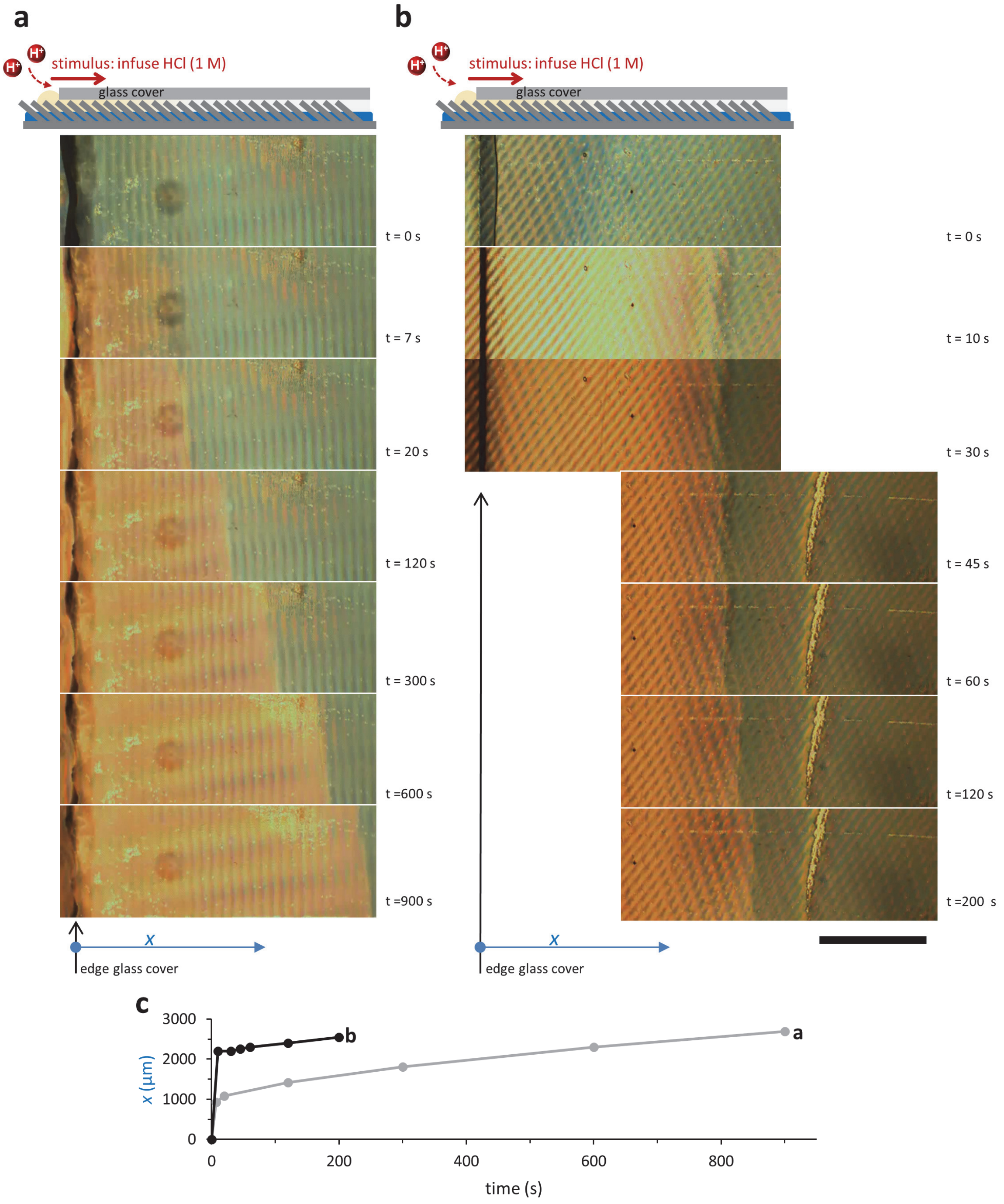

Supplementary Figure 5. Low magnification optical microscopy images visualizing the progression of Cu^{2+} release occurring upon diffusion of a 1 M HCl solution along a hydrogel-microplate substrate that is covered with a thin film of water and a glass slide (see schemes at top and Methods). **a, b,** Representative examples, revealing fast progression of the HCl front over the first 1 mm (a) or 2 mm (b), whereas further away from the edge of the glass cover the progression of the HCl front slows down. The scale bar equals 1 mm. **c,** Distance between the edge of the glass cover and the blue-to-colorless front (*x*) *vs*. time.

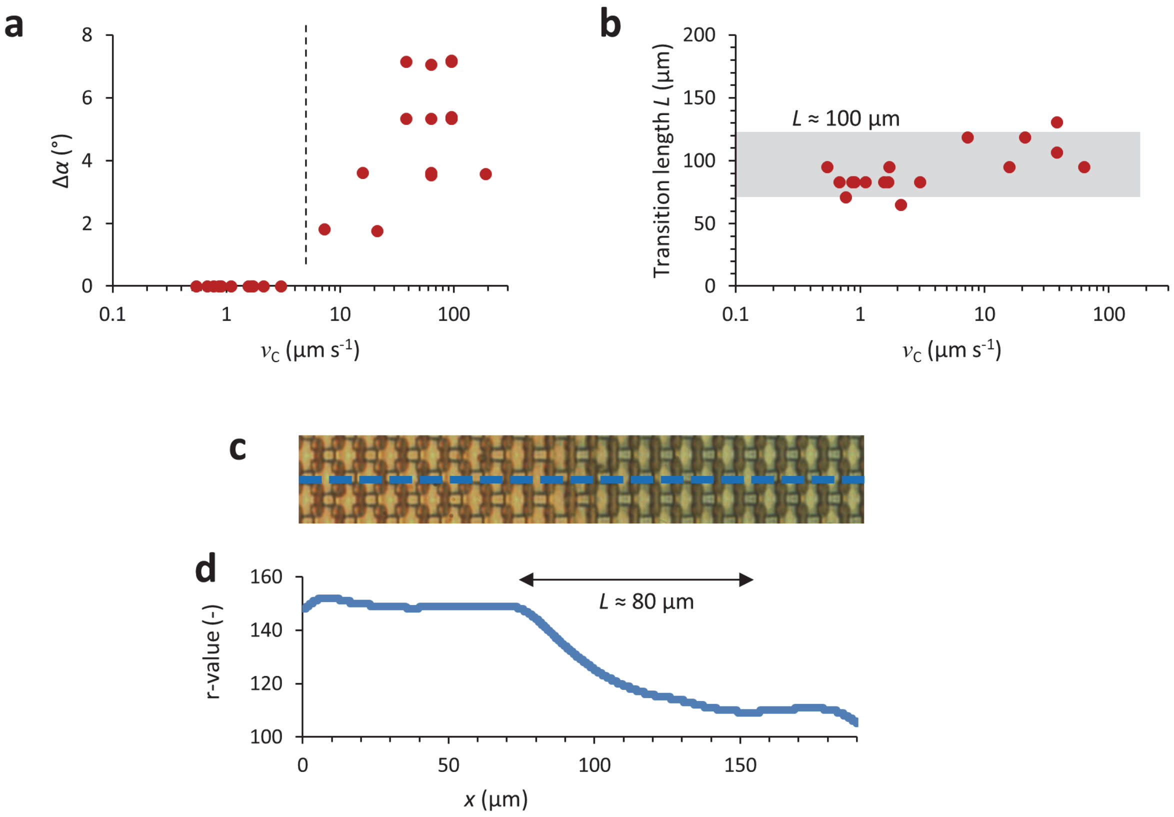

Supplementary Figure 6. a, Quantitative comparison of the hydrogel-microplate swelling/contraction pulse intensity ($\Delta\alpha$) at different color-transition progression speeds (v_C) of a 1 M HCl stimulus along a substrate containing stored Cu^{2+} (similar to the experiments shown in Fig. 4b-d of the main text; each dot represents a separate experiment). The pulse intensity $\Delta\alpha$ is defined as the absolute value of the difference between the minimum microplate tilt angle during the pulse and the microplate tilt angle before Cu^{2+} release (or after Cu^{2+} release, depending on which tilt angle gives the smallest $\Delta\alpha$ value). When no pulse is observed, $\Delta\alpha = 0$. The data in the graph further demonstrate that a swelling/contraction wave is obtained only above a threshold value of $v_C \approx 5 - 8$ µm s^{-1}. **b,** Transition length L *vs*. v_C, showing the typical length scale of the blue-to-colorless transition (as exemplified in **c** for the experiment shown in Fig. 4b of the main text, $v_C = 0.76$ µm s^{-1}). **d,** r-value *vs*. position x, acquired along the blue dashed line in (c).

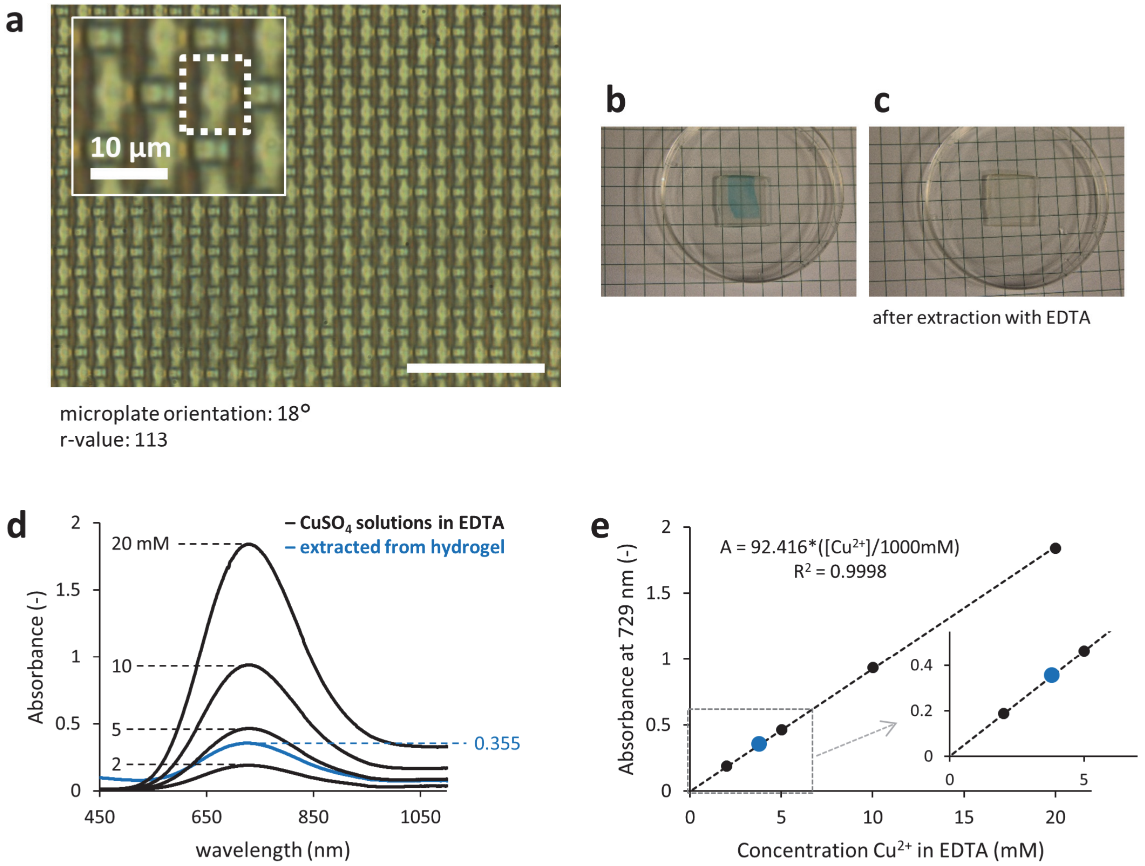

Supplementary Figure 7. a, Optical microscopy images of a hydrogel-microplate substrate that was sequentially pre-treated with 1 M HCl, water, 0.1 M KOH, water, contracted upon adding $CuSO_4$ (0.8 M, 100 µL), and rinsed with water (10x 1mL). The scale bar equals 50 µm. **b,c**, Photos of hydrogel-microplate substrates in a round petri dish, before (**b**) and after (**c**) extraction of Cu^{2+} (initially complexed to the hydrogel) with an ethylenediaminetetraacetate (EDTA) solution. To quantify the Cu^{2+} concentration in the contracted hydrogels, Cu^{2+} is extracted upon subsequent addition and removal of 10 volumes of 100 µL EDTA solution (0.27 M in 1 M KOH), resulting in the colorless, Cu^{2+}-free substrate shown in (c). **d,** Absorption spectra of EDTA-Cu^{2+} solutions, and the solution containing Cu^{2+} that was extracted from the hydrogel substrate in (b). **e,** The optical densities are compared to absorption spectra of aqueous EDTA solutions (0.27 M, 1 M KOH) with different $CuSO_4$ concentrations (black, 2 mM; 5 mM; 10 mM; 20 mM, full spectra shown in d). Based on the optical density of the Cu^{2+} extract from the hydrogel, the Cu^{2+} concentration is determined to be 3.8 mM, indicating that 0.0038 mmol Cu^{2+} was complexed to the hydrogel substrate.

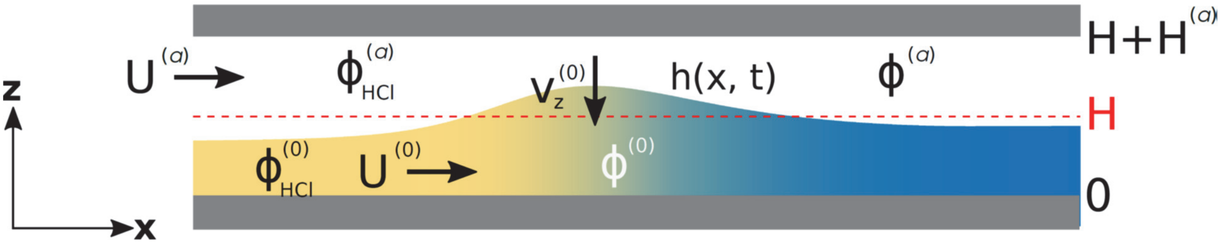

Supplementary Figure 8. Continuum model of mechanical wave progression: schematics. Our theoretical formulation considers a hydrogel layer with an equilibrium height H (shown by the red dashed line) and a supernatant domain with an equilibrium height $H^{(a)}$, which are enclosed by rigid boundaries (grey) at the top and the bottom, corresponding to the glass slide and the epoxy substrate in experiments, respectively. In a two-dimensional formulation (*i.e. xz*—plane), an acid stimulus injected into the channel with a speed $U^{(a)}$ (averaged over the height of the supernatant domain) transports a volume fraction field of the HCl (equivalently H^+) $\phi_{HCl}^{(a)}$, governed by Eq. 23. At the leading order $O(\delta^0, \epsilon^0)$, $\phi_{HCl}^{(a)}$ is carried into the hydrogel layer via diffusion, resulting in a volume fraction $\phi_{HCl}^{(0)}$, determined by Eq. 19. A fluid flow within the hydrogel with a speed $U^{(0)}$ emerges due to the gradients of the pressure $p^{(0)}$ (Eq. 3). When H^+ breaks the COO^--Cu^{2+}-COO^- complexes (yellow region) that store Cu^{2+} in the backbone with a volume fraction $\phi^{(b)}$ (not shown), free Cu^{2+} with a volume fraction $\phi^{(0)}$ creates a transient osmotic imbalance, resulting in a vertical flow $v_z^{(0)}$ (Eq. 14). H^+ protonates COO^- groups, equivalent to a storage of the corresponding volume fraction $\phi_{HCl}^{(b)}$ (not shown) in the backbone. The released Cu^{2+} diffuses into the supernatant layer at a time scale $H^2/D_{Cu^{2+}} \sim 10$ s, where $D_{Cu^{2+}}$ is the diffusion constant of Cu^{2+} ions inside the hydrogel, resulting in a non-zero volume fraction $\phi^{(a)}$ in the upper domain. The regions with a finite $\phi^{(b)}$ are depicted in blue. The height $h(x, t)$ deviates from its equilibrium value H because of a non-zero $v_z^{(0)}$, a pressure caused by $\phi^{(b)}$, and a pressure due to the hydrophobicity caused by $\phi_{HCl}^{(b)}$.

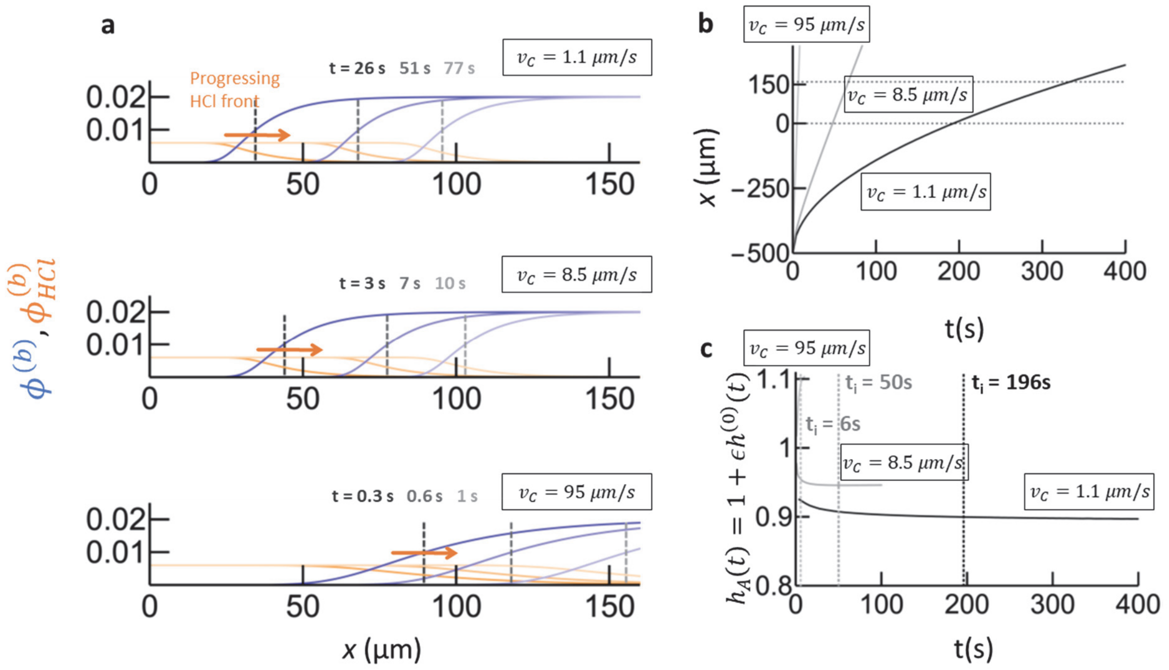

Supplementary Figure 9. Modeling of the dynamics of acid stimulus progression and subsequent Cu^{2+} release. **a,** For the inlet HCl injection speeds $U^{(a)} = 0.2$ s^{-1} (top), $U^{(a)} = 24$ s^{-1} (middle), $U^{(a)} = 250$ s^{-1} (bottom), the advancing acid along the hydrogel (x-direction) interacts with the carboxyls on the polymer backbone ($\phi^{(b)}_{HCl}$, orange) and in turn decomplexes Cu^{2+} ($\phi^{(b)}$, blue) from the gel. The theoretical blue-to-colorless transition progression speeds v_C – indicating release of Cu^{2+} from the gel – are calculated from data in b). The color scales change from dark to light with increasing time. The vertical dashed lines denoting the stimulus front are at the half of the maximum acid stimulus volume fraction $\phi^*_{HCl}/2$. Each of them corresponds to $t' \equiv t - t_i$, where t_i is taken as the time when the stimulus front has traversed 500 μm in real units along the domain (see b), so as to reach a quasi-steady state. The greyscale changes from dark to light with increasing t'; the values of t_i as a function of $U^{(a)}$ are given in c). The plots are associated with the swelling profiles shown in Fig. 4 in the main text. The full evolution of the curves in time is shown in Supplementary Movies 4-6. **b,** Position x of the mechanical wave as a function of the injection speed $U^{(a)}$ and time. The inlet is at $x = -500$ μm and the horizontal dotted lines enclose the position interval $x \in [0, 150\ \mu\text{m}]$ in which data from a) and Fig. 4 of the main text are shown. The progression speed of the color transition v_C is given by the slope of the curves within $x \in [0, 150\ \mu\text{m}]$. This calculation is based on the experimental observation that the progression of the mechanical wave and recession of the blue color occur with a constant phase difference. The theoretical inlet HCl injection speeds for the curves, from dark to light grey, are $U^{(a)} = 0.2$ s^{-1}, $U^{(a)} = 24$ s^{-1}, $U^{(a)} = 250$ s^{-1}. **c,** Amplitude h_A of the mechanical wave as a function of the injection speed $U^{(a)}$ and time. The dotted vertical lines correspond to t_i, which is defined as the time when the signal front has traversed 500 μm in real units along the domain, so as to reach a quasi-steady state. In (b) and (c), the greyscale of the curves changes from dark to light with increasing $U^{(a)}$.

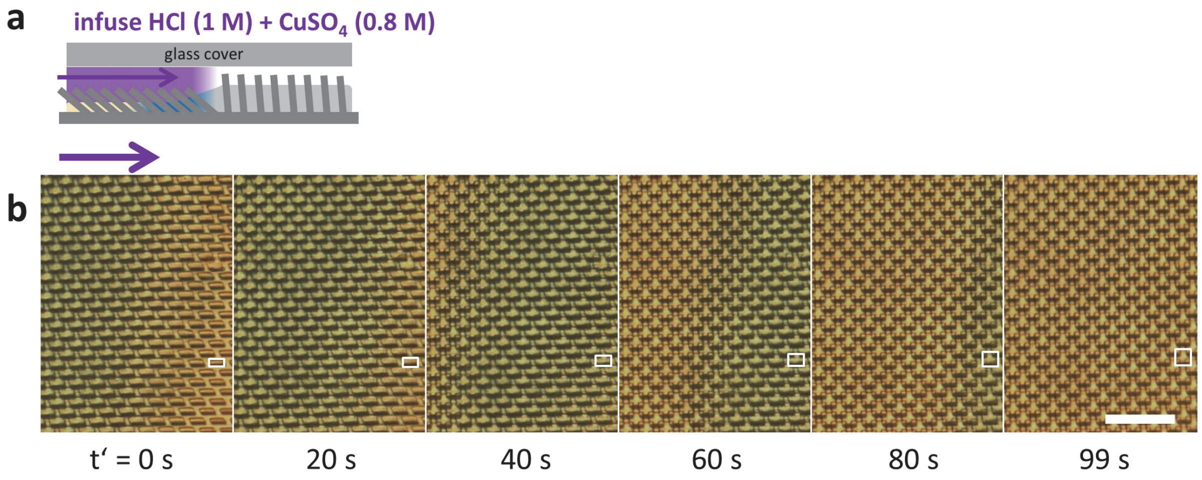

Supplementary Figure 10. a) An aqueous solution containing both HCl (1 M) and $CuSO_4$ (0.8 M) is guided along a hydrogel-microplate substrate with initially upright oriented microplates, covered with a thin layer of water and a glass slide, as indicated by the scheme at the top. For this experiment, a solution of 0.05 M KOH was used for the pre-treatment of the hydrogel. **b)** The optical microscopy images reveal a blue band in the front region of the progressing HCl/$CuSO_4$ solution, moving with a rate of $v_C = 3$ µm s^{-1} from left to right in the images. This observation indicates that the graded concentration of both Cu^{2+} and acid in the front allows a transient complexation of Cu^{2+} to the PAA hydrogel, prior to Cu^{2+} being overtaken and released by a saturating acid concentration of 1 M HCl. The scale bar equals 50 µm.

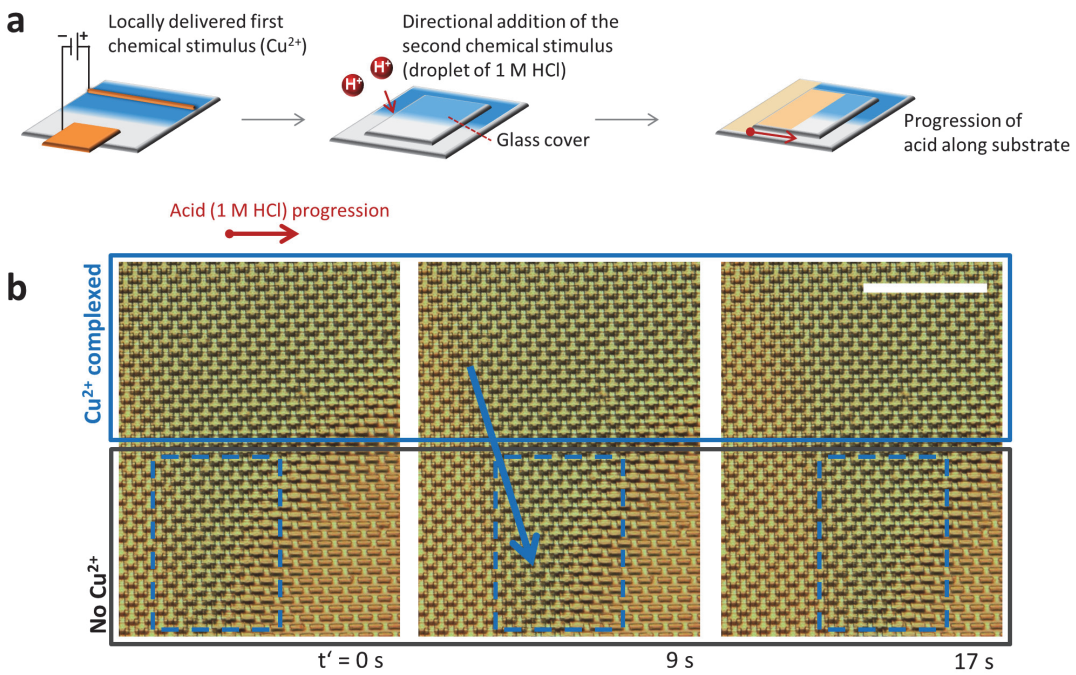

Supplementary Figure 11. a) Following electrochemical delivery of Cu^{2+} to the top region of the substrate, a glass cover was applied and the acid stimulus (1 M HCl) was allowed to progress from left to right along the substrate, in analogy to the experiments shown in Fig. 5 of the main text. **b)** The optical microscopy images reveal that upon progression of the acid with an intermediate rate of $v_C = 3$ µm s^{-1}, copper ions released at the acid front in the Cu^{2+}-complexing region (top region, indicated by the blue box) migrate to the adjacent region as indicated by the blue arrow, generating a transient blue wave – indicated by the blue dashed box – at the stimulus front in the region where Cu^{2+} was not initially complexed (bottom region, indicated by the grey box). Scale bar: 100 µm.

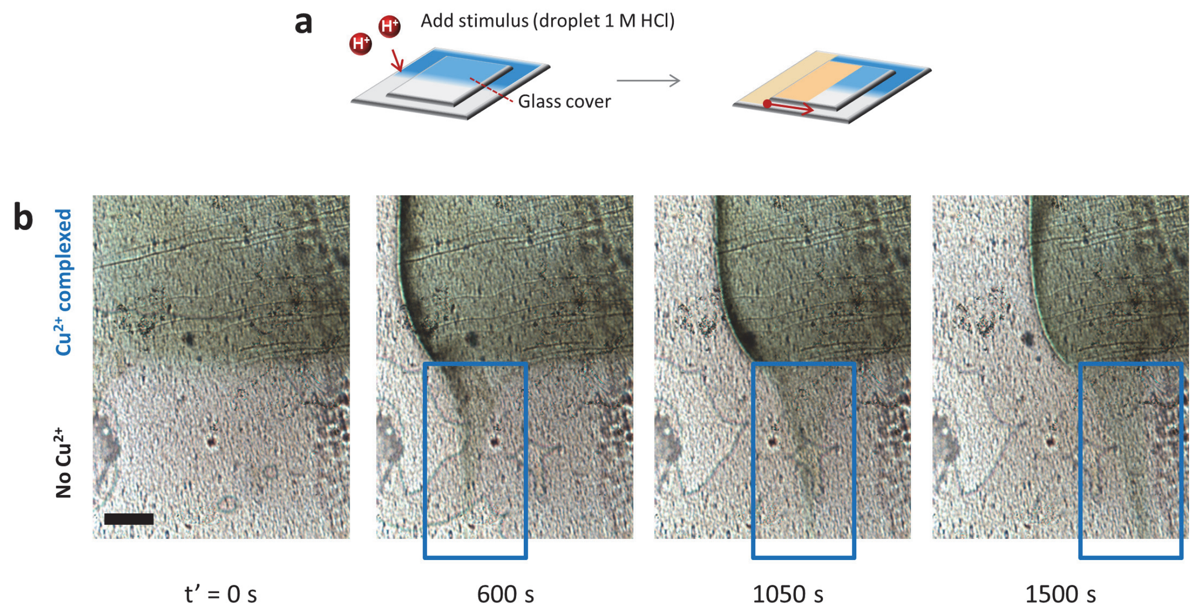

Supplementary Figure 12. a) Migration and re-complexation of released Cu^{2+} generates a transient blue wave on a hydrogel substrate with no microplates. In analogy to the experiments shown in Fig. 5 of the main text, 1 M HCl is added from the left and allowed to progress along a substrate that has Cu^{2+} complexed on one side (top half of images) and not the other (bottom half of images). **b)** Cu^{2+} is released from the Cu^{2+}-complexing region and migrates to the adjacent, initially Cu^{2+}-free region of the microscopy images, where a transient blue wave appears (indicated by the blue boxes) just ahead of the progressing HCl stimulus front. Scale bar: 500 µm; v_C = 1 µm s^{-1}.

Supplementary Discussion

To corroborate the framework that sets up hydrogel systems to selectively report stimuli with specific dynamic characteristics, we develop a continuum theory that couples the association/dissociation of the complexing agents, flow/concentration of the downstream stimuli, and the resulting mechanical response of the hydrogel. Our theory employs the Biot model of consolidation and elasticity for a porous material to quantify the mechanics of the hydrogel.[1-3] Specifically, we extend the thin-film theory of a poroelastic layer developed by Jensen *et al* (Supplementary Reference 4) by taking into account the flow of the supernatant solution that transports the chemical stimuli, which permeate into the hydrogel and interact with its backbone, giving rise to transient osmotic pressure change, hydrophobicity, and in turn swelling/contraction. The hydrogel-supernatant solution domain is schematized in Supplementary Fig. 8. Denoting $\vec{v}$ as the local velocity of the fluid relative to the solid matrix, $\vec{u}$ as the displacement vector of the solid, $\tilde{r}$ as the rate of complexation/decomplexation, ϕ_k and $\phi_k^{(b)}$, respectively, as the volume fraction of the species k free in the hydrogel and attached to its backbone, ϕ_{HCl}^{*} as the maximum HCl volume fraction (equivalent to H^+ volume fraction) that the backbone can accommodate, σ and $\varepsilon \equiv (\vec{\nabla}\vec{u} + \vec{\nabla}\vec{u}^T)/2$ as the stress and strain tensors, the conservation of mass and momentum inside the hydrogel are given by ($\partial_t \equiv \frac{\partial}{\partial t}$; t: time)

$$\vec{\nabla} \cdot (\vec{v} + \partial_t \vec{u}) = \tilde{r}\phi_{HCl}\phi^{(b)} - \tilde{r}\phi_{HCl}(\phi_{HCl}^{*} - \phi_{HCl}^{(b)})\,, \tag{1}$$

$$\vec{\nabla} \cdot \sigma(\varepsilon, t) = 0\,, \tag{2}$$

where $\vec{v}$ is given by the Darcy's law (p: pressure, k_f: hydraulic permeability of the hydrogel, μ_f: kinematic viscosity of the solvent) as

$$\vec{v} = -\frac{k_f}{\mu_f}\vec{\nabla} p\,, \tag{3}$$

and the components of the stress tensor are given by

$$\sigma_{ij} \equiv 2\mu\varepsilon_{ij} + \delta_{ij}\left(\lambda\varepsilon_{kk} - p + \chi\phi_{HCl}^{(b)} + \gamma\phi^{(b)}\right)\,. \tag{4}$$

Eq. 1 ignores the recomplexation of free Cu^{2+} in the hydrogel as this process plays no role in the dynamics of mechanical waves. In Eq. 4, $\mu, \lambda \sim 10^4 Pa$ are Lamé coefficients, χ, γ are free parameters, and $\phi^{(b)} \equiv \phi_{Cu^{2+}}^{(b)}$ for brevity. The last two terms of Eq. 4 describe hydrogel contraction due to an emergent hydrophobicity ($\phi_{HCl}^{(b)} > 0$) or to COO^--Cu^{2+}-COO^- complexation ($\phi^{(b)} > 0$). The osmosis-driven swelling behavior must be governed by a characteristic pressure scale $\bar{p} \equiv [Cu^{2+}]k_BT \sim 10^6$ Pa at room temperature, where $[Cu^{2+}]$ follows from the molar concentration of the free copper ions in the solution. Substituting Eq. 3 into Eq. 1 yields the diffusion constant of the water within the hydrogel as $D_{water} \equiv k_f\bar{p}/\mu_f$. When the height of the hydrogel-supernatant solution interface is defined by h, the solution to Eqs. 2 and 4 requires boundary conditions at $z = 0$ and $z = h(x,t)$ in a two-dimensional (2D) vertical cross-section of the hydrogel film (*i.e.* xz −plane, see Supplementary Fig. 8) which are given as

$$z = h; \quad \hat{n}\cdot\sigma\cdot\hat{n} = 0, \quad \hat{t}\cdot\sigma\cdot\hat{n} = 0, \tag{5}$$

$$\text{at} \quad z = 0; \quad \vec{u} = 0. \tag{6}$$

Here, $\hat{n}$ and $\hat{t}$ are the unit vectors at the interface in the normal and tangential direction, respectively, and are expressed as

$$\hat{n} \equiv \frac{1}{\sqrt{1+(\partial_x h)^2}}\left(-\frac{\partial h}{\partial x}\hat{\imath} + \hat{k}\right), \quad \hat{t} \equiv \frac{1}{\sqrt{1+(\partial_x h)^2}}\left(\hat{\imath} + \frac{\partial h}{\partial x}\hat{k}\right), \tag{7}$$

where $\hat{\imath}, \hat{k}$ are unit vectors in the x and z directions. The notation ∂_j corresponds to $\frac{\partial}{\partial x}$ or $\frac{\partial}{\partial z}$ for $j = x, z$. The normal and tangential stress balance in Eq. 5 imposes a stress-free interface, because in our experiments the maximum pressure scale of the supernatant solution $\bar{P}^a \sim 0.1\, Pa$ is negligibly smaller than all pressure scales within the hydrogel ($\bar{P}^a \ll \bar{p}$; $\bar{P}^a \ll \mu$; $\bar{P}^a \ll \lambda$). Eq. 6 ensures no deformation at the hydrogel-substrate boundary.

The fluid flow in the supernatant domain is governed by the vanishing divergence of the flow field $\vec{V} \equiv V_x\hat{\imath} + V_z\hat{k}$ (mass conservation) and the Stokes flow (momentum conservation), which are given by (P: pressure in the supernatant domain)

$$\vec{\nabla}\cdot\vec{V} = 0, \tag{8}$$

$$\frac{\partial P}{\partial x} = \mu_f \vec{\nabla}^2 V_x, \quad \frac{\partial P}{\partial z} = \mu_f \vec{\nabla}^2 V_z. \tag{9}$$

The flow and pressure fields in Eqs. 8 and 9 are subject to the following boundary conditions at the hydrogel-supernatant domain interface ($z = h$) and the glass slide ($z = H + H^{(a)}$)

$$\text{at} \quad z = h; \quad \vec{V} = \vec{v} + \partial_t \vec{u}, \tag{10}$$

$$\text{at} \quad z = H + H^{(a)}, \quad \vec{V} = 0. \tag{11}$$

Our model is based on thin-film approximation $\delta << 1$, where $\delta \equiv H/L \approx 0.1$ is the aspect ratio of the film. Based on the dimensions of the channel in experiments, we take the aspect ratio associated with the supernatant domain $\delta^{(a)} \equiv H^{(a)}/L$ as $\delta^{(a)} = \delta$. The equilibrium film thickness is $H \approx 10$ µm, and we take $L \approx 100$ µm in accordance with the domain along which traveling swelling/contraction waves are observed upon HCl stimulus progression (Fig. 4a-c). Defining the pressure scale $\bar{P}^{(a)} \equiv \mu_f\, \bar{U}^{(a)}/L$, scaling the lengths in x and z-directions by L and H, respectively, Eq. 9 in dimensionless form at $O(\delta^0)$ is given by Supplementary Reference 5

$$\frac{\partial P}{\partial x} = \frac{\partial^2 V_x}{\partial z^2}, \quad \frac{\partial P}{\partial z} = 0. \tag{12}$$

Next, we define the deformation ratio $\equiv a/H$, where a is the vertical deformation length. By evaluating the tangential stress balance at the interface (Eq. 5) in the limit $\delta \ll 1$, it can be shown that the horizontal displacement must be on the order of δa. Therefore, we define the dimensionless

components of the displacement vector as $u'_x \equiv \delta a u_x$ and $u'_z \equiv a u_z$ and then drop the primes. We scale the hydrogel pressure by $\bar{p}$, the flow velocity within the hydrogel by $U^{(0)} \equiv k_f \bar{p}/\mu_f L$, and the flow velocity within the supernatant domain by $\bar{U}^{(a)}$. Then, obeying the hierarchy between scales[4] $\delta^2 < \epsilon \ll 1$, we perform a series expansion of all dimensionless variables as follows:

$$\frac{h-1}{\epsilon} = h^{(0)} + \epsilon h^{(1)} + \mathcal{O}(\epsilon^2, \delta^2)\,, \tag{13a}$$
$$p = p^{(0)} + \epsilon p^{(1)} + \mathcal{O}(\epsilon^2, \delta^2)\,, \tag{13b}$$
$$u_j = u_j^{(0)} + \epsilon u_j^{(1)} + \mathcal{O}(\epsilon^2, \delta^2)\,, \tag{13c}$$
$$v_j = v_j^{(0)} + \epsilon v_j^{(1)} + \mathcal{O}(\epsilon^2, \delta^2)\,, \tag{13d}$$
$$\phi_k = \phi_k^{(0)} + \epsilon \phi_k^{(1)} + \mathcal{O}(\epsilon^2, \delta^2)\,. \tag{13e}$$
$$V_j = V_j^{(0)} + \epsilon V_j^{(1)} + \mathcal{O}(\epsilon^2, \delta^2)\,, \tag{13f}$$
$$P = P^{(0)} + \epsilon P^{(1)} + \mathcal{O}(\epsilon^2, \delta^2)\,. \tag{13g}$$

Although Eqs. 13f and 13g are not in the hydrogel phase, their series expansion is well-defined both at the hydrogel-supernatant solution interface (where Eq. 13a holds) and the entire solution domain. This is because, first, the pressure is constant in the vertical direction (Eq. 12), and, second, V_x, V_z are functions of the dimensionless boundary conditions at the interface (Eq. 10) across which flux continuity holds. Eq. 12 with the dimensionless form of the continuity equation (Eq. 8) yields the dimensionless components of $\vec{V}$ at $O(\delta^0, \epsilon^0)$ subject to the dimensionless boundary conditions at $z = 1$ (from Eq. 10) and $z = 2$ (from Eq. 11). Note that, in order to close the equations, we need to approximate the osmotic vertical flow to

$$v_z^{(0)} = Q_{osm}(p_{osm} - \tilde{P})\,, \tag{14}$$

(Supplementary Fig. 8), and

$$p^{(0)} \equiv \alpha h^{(0)} + \chi \phi_{HCl}^{(b)} + \gamma \phi^{(b)}\,, \quad p_{osm} \equiv p^{(0)} - \phi^{(0)}\,, \quad \tilde{P} = -\phi^{(a)}\,. \tag{15}$$

Here, the Cu^{2+} volume fraction in the supernatant solution is denoted by $\phi^{(a)}$. The constant α is derived from the relations between the Lamé coefficients, the Young's modulus E, and the Poisson's ratio ν in 2D. These relations are: $\mu \equiv E/2(1+\nu)$, and $\lambda \equiv E\nu/2(1-\nu^2)$, which yield $\alpha \equiv \frac{\epsilon E}{(1-\nu^2)\bar{p}}$. The coefficients χ and γ are free parameters. When the dominant term in p_{osm} is the free Cu^{2+} volume fraction within the hydrogel denoted by $\phi^{(0)}$, the mobility coefficient in Eq. 14 must then be in the order $Q_{osm} \sim \frac{A}{\delta^2}$. Here $A \equiv 1/1\,M\,\bar{\upsilon} \sim 10^2$ is the conversion coefficient ($\bar{\upsilon} = 10^{-26} L$: volume of a generic particle) since both p_{osm} and $\tilde{P}$ are expressed in terms of the volume fractions $\phi^{(0)}, \phi^{(a)}$ instead of molar concentration. In Eq. 15, $p^{(0)}$ is obtained by solving Eqs. 2 and 4 subject to the (i) boundary conditions in Eqs. 5 and 6 at $O(\delta^0, \epsilon^0)$, (ii) the series expressions given in Eq. 13, and (iii) a linear relation[3] between the interface height $h^{(0)}$ and the vertical deformation component $u_z^{(0)}$

$$u_z^{(0)} = h^{(0)} z\,, \tag{16}$$

which obeys Eq. 6 and is in accordance with Eq. 13a. The characteristic time of the hydrogel deformations in the vertical direction is $\tau_\perp \equiv \epsilon L/U^{(0)}$, where $U^{(0)} \equiv \frac{k_f \bar{p}}{\mu_f L} \approx 1$ s^{-1} is the velocity scale inside the hydrogel. In terms of the vertical deformation time $\tau_\perp \equiv \epsilon L/U^{(0)}$, the diffusion time of deformations in the horizontal direction is given by $\tau_\parallel \equiv \frac{\bar{p}\,(1-\nu^2)}{E}\tau_\perp$. Because $E/\bar{p} \ll 1$, vertical swelling must diffuse gradually. To account for the corresponding diffusive relaxation dynamics of $h^{(0)}$ to zero when $\phi_{HCl}^{(b)} \to 0$ and $\phi^{(0)} \to \phi^{(a)}$, we retain the term $\alpha\, h^{(0)}$ in $p^{(0)}$ (Eq. 15), although $\alpha < O(\epsilon)$. This is because the only pressure scale in a hydrogel without free species is set by E. Defining the dimensionless time $t' \equiv \tau_\perp t$ and dropping the prime, the height of the hydrogel at order $O(\delta^0, \epsilon^0)$ is governed by the dimensionless equation,

$$\frac{\partial h^{(0)}}{\partial t} = \frac{\partial^2 p^{(0)}}{\partial x^2} - Q_{osm}(p_{osm} - \tilde{P})\,, \tag{17}$$

which is obtained by averaging the dimensionless combination of Eqs. 1 and 3 over the hydrogel height and using Eqs. 14-16. We assume that the right-hand side of Eq. 1 is at $O(\epsilon)$, meaning that the simultaneous complexation of H^+ and decomplexation of Cu^{2+} alone do not lead to a local change of mass, hence no swelling/contraction of the hydrogel at the leading order. As a result, the only source term in Eq. 17 is governed by the osmotic pressure. The second term on the right-hand side of Eq. 17 contains a term proportional to $\phi^{(0)} - \phi^{(a)}$ (see Eq. 15), which must create a downward osmotic flow only when $\phi^{(0)} > \phi^{(a)}$. An upward osmotic flow from the dense hydrogel domain to the aqueous domain is not possible when $\phi^{(0)} < \phi^{(a)}$. To take it into account we modify $(\phi^{(0)} - \phi^{(a)}) \to (\phi^{(0)} - \phi^{(a)}) \times f(\phi^{(0)} - \phi^{(a)})$, where $f(x) = [1 + \tanh(\Lambda\, x)]/2$ and $\Lambda \gg 1$. This step function suppresses the spurious upward osmotic effect when $\phi^{(0)} < \phi^{(a)}$.

Next, we close the Eqs. 15 and 17 by introducing the dimensionless height-averaged mass conservation equations for the free species in the hydrogel and the supernatant domain, as well as the species attached to the polymer backbone. By defining the Peclet number $Pe \equiv U^{(0)}L/D^{(a)}$ ($D^{(a)} \approx 10^{-9}$-10^{-10} m^2 s^{-1} the diffusivity of ions in the supernatant solution) and $r \equiv \tilde{r}\tau_\perp$ as the dimensionless rate of complexation/de-complexation to/from the backbone, the dimensionless continuity equations inside the hydrogel at the leading order become:

$$\frac{\partial \phi^{(0)}}{\partial t} = -\frac{\epsilon}{Pe\delta^2}(\phi^{(0)} - \phi^{(a)}) + r\phi_{HCl}^{(0)}\phi^{(b)}\,, \tag{18}$$

$$\frac{\partial \phi_{HCl}^{(0)}}{\partial t} = -\frac{\epsilon}{Pe\delta^2}(\phi_{HCl}^{(0)} - \phi_{HCl}^{(a)}) - r\phi_{HCl}^{(0)}(\phi_{HCl}^{*} - \phi_{HCl}^{(b)})\,. \tag{19}$$

The first terms on the right-hand side of both equations model the vertical diffusive flux between the hydrogel and the supernatant solution. At order $O(\delta^0, \epsilon^0)$ there is no contribution of advection and diffusion terms to the dynamics of free ions in the hydrogel in Eqs. 18 and 19. The dimensionless rate equations on the backbone are given as

$$\frac{\partial \phi^{(b)}}{\partial t} = -r\phi_{HCl}^{(0)}\phi^{(b)} \,, \tag{20}$$

$$\frac{\partial \phi_{HCl}^{(b)}}{\partial t} = r\phi_{HCl}^{(0)}(\phi_{HCl}^{*} - \phi_{HCl}^{(b)}) \,. \tag{21}$$

Defining $b \equiv U^{(0)}/U^{(a)}$, where $U^{(a)} \sim$ 0.2-250 μm s^{-1} is the theoretical injection speed of the HCl stimulus, the dimensionless continuity equations in the supernatant solution at the leading order are given by

$$\frac{\partial \phi^{(a)}}{\partial t} + \frac{\epsilon}{b}\frac{\partial \phi^{(a)}}{\partial x} - \frac{\epsilon}{Pe}\frac{\partial^2 \phi^{(a)}}{\partial x^2} = \frac{\epsilon}{Pe\delta^2}(\phi^{(0)} - \phi^{(a)}) \,, \tag{22}$$

$$\frac{\partial \phi_{HCl}^{(a)}}{\partial t} + \frac{\epsilon}{b}\frac{\partial \phi_{HCl}^{(a)}}{\partial x} - \frac{\epsilon}{Pe}\frac{\partial^2 \phi_{HCl}^{(a)}}{\partial x^2} = \frac{\epsilon}{Pe\delta^2}(\phi_{HCl}^{(0)} - \phi_{HCl}^{(a)}) \,. \tag{23}$$

In these two equations, the second and third terms on the left-hand side correspond to the advection and horizontal diffusion of the chemicals, respectively. The advection terms are derived under the constraint

$$1 = \int_1^2 V_x^{(0)} dz + \int_0^1 v_x^{(0)} dz \,, \tag{24}$$

which ensures flux continuity along the supernatant domain, provided that the left-hand side is the normalized flux through the inlet at $O(\delta^0, \epsilon^0)$. Eqs. (15), (17), (18)-(23) are solved in a domain $x \in [0,10]$ subject to the boundary conditions

$$\text{at} \quad x = 0\,; \quad \frac{\partial p}{\partial x} = 0\,, \quad \phi_{HCl}^{(a)} = \phi_{HCl,i}\,, \quad \phi^{(a)} = \phi^{(0)}\,, \tag{25}$$

$$\text{at} \quad x = 10\,; \quad \frac{\partial p}{\partial x} = 0\,, \quad \frac{\partial \phi_{HCl}^{(a)}}{\partial x} = 0\,, \quad \frac{\partial \phi^{(a)}}{\partial x} = 0\,, \tag{26}$$

where $x = 10$ corresponds to the far field with pure fluid flow, no free species and no deformations. Here, $\phi_{HCl,i} = \phi_{HCl}^*$, namely the HCl volume fraction at the inlet is taken equal to the critical HCl volume fraction. In Eqs. (25) and (26), $\partial p/\partial x = 0$ ensures that the fluxes at the inlet and outlet are only through the cross-section of the supernatant domain.
We take the initial conditions as

$$\begin{aligned} &h^{(0)} = h_i = -1\,, \quad \phi_{HCl}^{(a)} = \phi_{HCl,i}e^{-x/0.01}\,, \\ &\phi^{(a)} = \phi^{(0)} = 0\,, \quad \phi_{HCl}^{(0)} = \phi_{HCl}^{(b)} = 0\,, \quad \phi^{(b)} = \phi^*\,, \end{aligned} \tag{27}$$

where $\phi^* = 0.02$ is equivalent to 2.9 M of Cu^{2+} in water, and $\phi_{HCl}^* = 0.006$ corresponds to 1 M of HCl in water. Eqs. (15), (17), (18)-(23), (25)-(27) constitute a time-dependent boundary value problem of coupled nonlinear equations, which we numerically solve using the COMSOL finite element package.[6] We determine the free parameters (i) χ, (ii) γ in Eq. 15 by imposing $p_{osm} -$

$\tilde{P} = 0$ when $\phi^{(0)} = \phi^{(a)}$ (vanishing osmotic flow; see Eq. 17), when (i) $\phi^{(b)} = 0$, and (ii) $\phi_{HCl}^{(b)} = 0$, which yield the following relations:

$$\chi = -\frac{\alpha h_{i,HCl}}{\phi_{HCl}^{*}}, \quad \gamma = -\frac{\alpha h_i}{\phi^{*}}, \tag{28}$$

In accordance with the experimental measurements and material parameters, we choose the other unknowns as: $r = 5 \times 10^4, \epsilon = 0.1,\ Pe = 0.1, \delta = 0.1,\ \alpha = 0.001,\ Q_{osm} = 10^2/\delta^2,\ h_i = -1, h_{i,HCl} = -1.2$.

The value of k_f can be approximated via the concentration of COO^--Cu^{2+}-COO^- crosslinks in the contracted hydrogel; based on a Cu^{2+} concentration of 2.9 M (see Supplementary Fig. 7), we obtain a volume of 0.57 nm^3 per crosslink, *i.e.* $k_f \approx 10^{-19}$-10^{-18} m^2. Using $k_f \approx 10^{-19}$ m^2 and $\mu_f = 10^{-3}$ Pa s, the vertical diffusion constant of water is given as $D_{water} \equiv \frac{k_f \bar{p}}{\mu_f} \sim 10^{-10}$ m^2 s^{-1}.

The theoretical results are summarized in Fig. 4e, Supplementary Figs. 8 and 9, and Supplementary Movies 4-6. The computations are performed in dimensionless units, and the figure axes are then converted to real units for comparison with experiments. In our simulations $b \equiv U^{(0)}/U^{(a)}$ is a fitting parameter to determine the best matching values of the blue-to-colorless progression speed v_C to the experiments. When the free Cu^{2+} production rate in the hydrogel is bigger than the rate of local hydrogel deformations, equivalent to $v_c/L > 1/\tau_\perp$, *i.e.* $\epsilon v_c/U^{(0)} > 1$, then osmotic swelling must occur when $v_c > 10$ μm s^{-1}. This inequality does not hold at sufficiently low HCl injection rates. As a result, there is no transient swelling as in b = 5 ($U^{(a)} = 0.2$); the propagation of H^+ association to the backbone and the front of subsequent Cu^{2+} decomplexation from the backbone (Supplementary Fig. 9a, top) lead to an advancing collapse of the hydrogel (Fig. 4b, Supplementary Movie 4). When the production rate of free Cu^{2+} in the hydrogel increases upon faster HCl injection, then osmotic swelling can temporally dominate over mechanical collapse due to protonation of the hydrogel, generating traveling deformation waves at b = 0.041 ($U^{(a)} = 24\ s^{-1}$) and b = 0.004 ($U^{(a)} = 250$ μm s^{-1}) (Fig. 4c and 4d, respectively, Supplementary Figs. 9a middle and bottom, respectively, and Supplementary Movies 5 and 6). The swelling wave speed (Supplementary Fig. 9b) and the amplitude (Supplementary Fig. 9c), as well as the wavelength, increase as the free Cu^{2+} migrating into the supernatant domain is washed out faster with an increasing HCl injection rate, leading to a longer osmotic imbalance between the gel and the supernatant phase across the channel, as well as an augmented osmotic flow. At b = 0.041 and b = 0.004, the wavelength is on the order of 50 μm (Fig. 4c) and >200 μm (Fig. 4d), respectively. The dynamics of Cu^{2+} decomplexation and H^+ association fronts are similar at b = 5; 0.041; 0.0004, yet the progression speed naturally increases commensurate with the HCl injection rate (Supplementary Fig. 9b, Supplementary Movies 4-6).

In summary, our theoretical model quantitatively reproduces the conditions for mechanical swelling/deswelling wave formation selective for fast acid stimulus injection rates. Furthermore, the length- and time scales associated with the wave propagation dynamics due to osmotic imbalance are at the same orders of magnitude with our experimental observations.

Supplementary References

1. Biot MA (1941) *J. Appl. Phys.* 12:155-164.
2. Biot MA (1956) *J. Appl. Mech.* 78:91-96.
3. Biot MA, Willis DG (1957) *J. Appl. Mech.* 24:594-601.
4. Jensen OE, Glucksberg MR, Sachs JR, Grotberg JB, *J. Appl. Mech.* 61:726-728.
5. Oron A, Davis SH, Bankoff SG, *Rev. Mod. Phys.* 69:931-980.
6. See http://www.comsol.com for COMSOL 5.0a, Burlington, MA, USA.
