# Supplementary material for: Non-equilibrium signal integration in hydrogels
Source: Nat Commun. 2020 Jan 20;11:386. doi: 10.1038/s41467-019-14114-0 (PMC6971035; doi:10.1038/s41467-019-14114-0)
Supplement: Supplementary file 2 — Description of Additional Supplementary Files [file 41467_2019_14114_MOESM2_ESM.pdf]

Description of Additional Supplementary Files

File Name: Supplementary Movie 1

Description: Optical microscopy movie corresponding to Fig. 2e; upon applying a current of 0.1 mA (voltage approx. 1 V) to the copper electrode, which is mounted directly on top of the hydrogel-microplate substrate covered with a thin layer of an aqueous sodium perchlorate ($NaClO_4$, 0.05 M, 100 μL) electrolyte solution, the region of tilted microplates expands toward the right as the Cu^{2+} migrates from left to right over the substrate.

File Name: Supplementary Movie 2

Description: Optical microscopy movies corresponding to Fig. 3b and 3c. (left) Adding hydrochloric acid (HCl, 1 M) to a substrate containing complexed Cu^{2+} causes the microplates to stand upright briefly and subsequently tilt back toward the substrate. (right) Adding hydrochloric acid (HCl) solutions with progressively increasing concentrations to a substrate containing complexed Cu^{2+} leads to a slower release rate of Cu^{2+}, and therefore no osmotic swelling of the hydrogel occurs and the microplates remain tilted. The movie shows the release of Cu^{2+}, indicated by the loss of blue color, during the addition of 0.05 M HCl.

File Name: Supplementary Movie 3

Description: Optical microscopy movies corresponding to Fig. 4b-d. (left) Slow progression of HCl along the substrate (v_C = 0.76 μm s^{-1}) containing complexed Cu^{2+} yields release of Cu^{2+} at the stimulus front – indicated by the color transition – without a swelling/contraction pulse. (middle) Progression of HCl at an intermediate rate (v_C = 8.6 μm s^{-1}) along a substrate containing complexed Cu^{2+} yields a wave of weakly up-and-down moving microplates traveling at the front of the color transition. (right) Fast progression of HCl along the substrate ($v_C \geq$ 95 μm s^{-1}) containing complexed Cu^{2+} yields a high-amplitude pulse concomitant to the release of Cu^{2+}.

File Name: Supplementary Movie 4

Description: At $b \equiv U^{(0)}/U^{(a)} = 5$, the evolution of mechanical contraction (top) in response to the acid stimulus progression (bottom, orange curve) and subsequent Cu^{2+} release (bottom, blue curve) along the dimensionless hydrogel film position x/L, where $L = 10^{-4}$ m. The red dashed line depicts the relative hydrogel film height in the Cu^{2+}-complexed region. In the movie, the theoretical curves Fig. 4b and Supplementary Fig. 9a (top) are taken from the dimensionless interval $x \in [5, 6.5]$.

File Name: Supplementary Movie 5

Description: At $b \equiv U^{(0)}/U^{(a)} = 0.041$, the evolution of a mechanical wave with a well-defined amplitude (top) in response to the acid stimulus progression (bottom, orange curve) and subsequent Cu^{2+} release (bottom, blue curve) along the dimensionless hydrogel film position x/L, where $L = 10^{-4}$ m. The red dashed line depicts the relative hydrogel film height in the Cu^{2+} complexed region. In the movie, the theoretical curves Fig. 4c and Supplementary Fig. 9a (middle) are taken from the dimensionless interval $x \in [5, 6.5]$.

File Name: Supplementary Movie 6

Description: At $b \equiv U^{(0)}/U^{(a)} = 0.004$, the evolution of a mechanical wave with a well-defined amplitude (top) in response to the acid stimulus progression (bottom, orange curve) and subsequent Cu^{2+} release (bottom, blue curve) along the dimensionless hydrogel film position x/L, where $L = 10^{-4}$ m. Within the experimentally observed domain (Fig. 4), the wave acts as a pulse because the wavelength is bigger than the length of the domain. The red dashed line depicts the relative hydrogel film height in the Cu^{2+} complexed region. In the movie, the theoretical curves Fig. 4d and Supplementary Fig. 9a (bottom) are taken from the dimensionless interval $x \in [5, 6.5]$.

File Name: Supplementary Movie 7

Description: Optical microscopy movie corresponding to Fig. 5b and 5c. (left) Slow progression of HCl (1 M) along the substrate ($v_C = 1$ µm s^{-1}) with Cu^{2+} stored on one half results in release of Cu^{2+} ions th at migrate and transiently bind to the other half, generating a blue color wave that travels at the stimulus fr ont. (right) Fast progression of HCl (1 M) along the substrate ($v_C = 225$ µm s^{-1}) initiates an osmotic swelling/contraction wave at the stimulus front in the region of the substrate that contains stored Cu^{2+} ions that were electrochemically delivered.
